# Similar Virulence Gene Repertoires but Distinct Stress Tolerance and Pathogenicity-Associated Phenotypes in Representative *Salmonella* Typhimurium ST19 and ST213 Strains from Mexico

**DOI:** 10.3390/microorganisms14081854

**Published:** 2026-08-20

**Authors:** Flor Alexia Esquivel-Barriga, Gerardo Vázquez-Marrufo, Adrián Gómez-Baltazar, Andrea Monserrat Negrete-Paz, Carlos Torres-Vega, Manuel López-Rodríguez, Elda Araceli Hernández-Díaz, Ma. Soledad Vázquez-Garcidueñas

**Affiliations:** 1 División de Estudios de Posgrado, Facultad de Ciencias Médicas y Biológicas “Dr. Ignacio Chávez”, Universidad Michoacana de San Nicolás de Hidalgo, Morelia 58020, Michoacán, Mexico; flor.alexia.esquivel@umich.mx (F.A.E.-B.); andrea.negrete@umich.mx (A.M.N.-P.); 0617452b@umich.mx (E.A.H.-D.); 2 Centro Multidisciplinario de Estudios en Biotecnología, Facultad de Medicina Veterinaria y Zootecnia, Universidad Michoacana de San Nicolás de Hidalgo, Morelia 58893, Michoacán, Mexico; gerardo.marrufo@umich.mx; 3 Departamento de Investigación y Posgrado en Alimentos, Facultad de Química, Universidad Autónoma de Querétaro, Querétaro 76010, Querétaro, Mexico; adrian.gomez@uaq.mx; 4 Laboratorio de Histología, Facultad de Ciencias Médicas y Biológicas “Dr. Ignacio Chávez”, Universidad Michoacana de San Nicolás de Hidalgo, Morelia 58020, Michoacán, Mexico; carlos.torres@umich.mx; 5 Facultad de Medicina Veterinaria y Zootecnia, Universidad Michoacana de San Nicolás de Hidalgo, Morelia 58893, Michoacán, Mexico; manuel.lopez@umich.mx

**Keywords:** *Salmonella* Typhimurium, ST213, gastrointestinal stress, virulotype, *Caenorhabditis elegans*

## Abstract

Foodborne illnesses caused by *Salmonella* Typhimurium remain a major public health concern worldwide. Although ST19 has historically been a dominant lineage within this serotype, ST213 has become increasingly prevalent in Mexico. The biological factors underlying this epidemiological shift remain incompletely understood. In this study, we compared virulence-associated gene repertoires and stress-related phenotypes in representative *S.* Typhimurium ST19 and ST213 strains with distinct virulotypes (VTs). Comparative genomic analysis identified 119 virulence-associated genes distributed across 26 VTs, with most genes broadly conserved between genotypes. Representative strains were evaluated under simulated gastrointestinal tract (GIT) stress conditions, in post-stress recovery assays, and in a *Caenorhabditis elegans* infection model. Under the experimental conditions evaluated, the representative ST213 strains SAL109 (VT1), SAL115 (VT1), and SAL016 (VT12) tended to show higher persistence under host-associated stress conditions and greater intestinal colonization capacity in *C. elegans* than the ST19 strain SAL004 (VT23). However, strains sharing the same VT did not necessarily exhibit similar phenotypes, indicating that virulence-associated gene repertoires alone do not fully explain stress tolerance or host colonization behavior. Overall, these findings highlight phenotypic variability among strains with similar virulence gene content and support the importance of integrating genomic and phenotypic approaches to better understand the biology of emerging *S.* Typhimurium lineages.

## 1. Introduction

Diarrheal diseases caused by contaminated food or water affect about 550 million people each year and result in approximately 420,000 deaths worldwide, with children under five being the most affected group [1,2,3]. *Salmonella enterica* is a leading cause of diarrhea globally [4]. Salmonellosis poses a major public health challenge, with nearly 94 million cases and at least 155,000 deaths reported worldwide [5]. It also has a significant socioeconomic impact in both developing and developed countries [6]. Salmonellosis is an acute gastrointestinal infection, and the distribution of causative serotypes varies by region, with Enteritidis and Typhimurium the most common [7]. In recent decades, epidemiological research on *S. enterica* has shifted from serotype identification to genotyping, using methods such as Multilocus Sequence Typing (MLST) and, more recently, whole-genome comparative analysis (WGCA) [8,9,10].

MLST analyses have identified sequence type (ST) 19 as the ancestral lineage within the Typhimurium serotype and as one of the most common genotypes worldwide at the start of this century [8,11,12]. However, in recent decades, the replacement of ST19 by other STs has been documented globally [13,14,15,16,17]. In Mexico, *S.* Typhimurium is the most frequently isolated from both clinical samples and food. It has been shown that ST19 is the ancestral genotype within serotype Typhimurium and that ST213 has gradually overtaken it in Mexico. The shift from mainly ST19 isolates to ST213 was observed over a short sampling period early this century [18]. So far, the genomic and functional features behind this shift, as well as its public health implications, remain poorly understood [19,20].

A complementary typing method to MLST and WGCA is virulotyping, which classifies isolates by their virulence gene profiles, assigning the same virulotype (VT) to isolates that share all examined genes [21]. Virulotyping can provide functional insights into the pathogenic potential of bacterial lineages and may help explain why certain genotypes become epidemiologically dominant. VTs were originally identified using PCR assays targeting specific virulence genes, but whole-genome analysis of virulence genes enables identification of the complete set of virulence factors in a strain [22,23]. In addition to assessing the severity of host damage during infection, multiple studies have shown that virulence genes also help strains resist various stressors, including acidic pH, osmotic stress, oxidative stress, and starvation [24,25,26].

Over time, various animal models have been proposed and evaluated to study infections caused by non-typhoidal *Salmonella* serotypes, including the nematode *Caenorhabditis elegans*, zebrafish, mouse, guinea pig, chicken, calf, pig, and Rhesus macaque [27,28]. Each of these models has well-documented advantages and disadvantages, and among them, only the last three develop *S. enterica* gastrointestinal infections naturally, most closely resembling those in humans. Furthermore, the ethical framework of the 3Rs (Replacement, Reduction, Refinement) reflects current trends in the study of human diseases using animal models [29]. One way to apply the 3Rs principle is to use animal models that, based on current knowledge, are not considered to pose ethical concerns regarding pain, such as the nematode *C. elegans*.

Because *S*. Typhimurium can colonize and replicate in the intestine of the hermaphrodite nematode *C. elegans* [30], this organism is considered a valuable biological model for providing preliminary insights into host–pathogen interactions. Despite this model not allowing the study of the ecological role of *S.* Typhimurium in the gastrointestinal tract, and being physiologically rather distant from humans, it offers important advantages, including a short life cycle, rapid reproduction, body transparency that allows visualization of colonization by microscopy, and structural and functional similarities of its intestinal cells and immune system to those of humans [31,32]. Therefore, this model provides an experimental platform to evaluate differences in virulence and host colonization among *S.* Typhimurium serotypes and genotypes [33,34].

Although the epidemiological replacement of ST19 by ST213 in Mexico has been documented, the biological factors that may contribute to this shift remain incompletely understood. Bioinformatic and experimental research suggest that ST213 may exhibit enhanced virulence traits compared to ST19 [35,36], and since its initial discovery, ST213 has expanded globally [37]. Previous studies indicated that the displacement of ST19 by ST213 could be due to ST213’s superior adaptation to environmental and host-related stressors [38]. However, the relationship between virulence-associated gene repertoires and stress-related phenotypes in these genotypes remains unclear.

Therefore, this study comparatively evaluated representative *S*. Typhimurium ST19 and ST213 strains with distinct virulotypes under simulated gastrointestinal tract (GIT) stress conditions and in a *C. elegans* infection model. By integrating genomic and phenotypic approaches, this work aimed to determine whether strains with similar virulence-associated gene repertoires differ in stress tolerance, recovery, and host colonization capacity.

## 2. Materials and Methods

### 2.1. Retrieval of Genomes from Public Databases

Genomes of *S.* Typhimurium, related to genotypes ST19 and ST213 and isolated in Mexico, were retrieved from the EnteroBase platform. Among the genomes reported up to 10 October 2025, 92 ST213 isolates and 76 ST19 isolates were randomly selected. The number of genomes chosen reflected the availability of high-quality genomes for each genotype in EnteroBase. Of these 168 genomes, 30 were excluded for the following reasons: (i) in 10 cases, the year of isolation was unknown, and (ii) in 20 cases, there were no assembled genomes or raw reads. A total of 102 pairs of raw reads were obtained from the EMBL-EBI repository (European Bioinformatics Institute, Hinxton, UK), while the 36 assembled genomes (draft genomes) were downloaded directly from EnteroBase. Metadata for all ST19 strains selected for analysis are listed in Supplementary Table S1. Metadata for the ST213 strains not included in the previous study by Hernández-Díaz et al. [39] are also provided.

### 2.2. Genome Sequencing of New Mexican Strains of S. Typhimurium

Genomes of 11 *S*. Typhimurium strains isolated in Michoacán between 2008 and 2011 were obtained from animal-origin foods, mainly beef and pork. These strains were provided by the Michoacán State Public Health Laboratory (LESPM) in Morelia, Mexico. They were selected after confirming their serotype as Typhimurium through serotyping according to the Kauffman-White scheme and assigning ST using MLST based on seven loci (Table 1). The complete genomes of the 11 strains were sequenced by CD Genomics (formerly GeneWiz; Shirley, NY, USA) using paired-end technology (250-bp reads) on the Illumina HiSeq platform (Illumina, Inc., San Diego, CA, USA) and deposited in GenBank under BioProject ID PRJNA802840. As a reference for the analysis, the genome of strain ATCC14028 was used, with GenBank ID CP001363.1.

### 2.3. Bioinformatic Analysis

For the genomic analysis, 102 raw read sets retrieved from public databases (EMBL-EBI), 11 sets generated in this study, and 36 assembled genomes from Enterobase were included. Raw reads were processed with the Bactopia pipeline v.3.2.0 [40], which performed quality control, de novo assembly (SPAdes), and annotation (Prokka). Assembly quality of all 149 draft genomes, including those obtained from Enterobase, was assessed with QUAST (ver 5.0.2), integrated into the same pipeline, using the following metrics: genome size (~4.8–5.5 Mb), N50, number of contigs (≤250), average contig size, and largest contig length.

During this filtering process, three genomes from ST19 strains were removed because they exceeded the allowable contig limit. Quality metrics for the assembled genomes, from both our data and databases, are listed in Supplementary Table S2 for ST19 strains. For ST213, only data for strains whose genomes have not been previously reported by our research group [39] are included.

### 2.4. Analysis of Virulence-Associated Genes

Virulence-related genes were identified by screening the annotated genomes against the Virulence Factor Database (https://www.mgc.ac.cn/VFs/main.htm, accessed on 31 October 2025) [41] using ABRicate (ver 0.9.8), as implemented in the Bactopia pipeline. Hits with ≥80% nucleotide identity and ≥80% query coverage were considered positive. Only virulence genes annotated as “COMPLETE” (full-length gene sequences without truncations) in each genome were included in further analysis, whereas those annotated as “PARTIAL” (incomplete or truncated sequences) were excluded. A binary matrix (1/0) was constructed from the identified genes to indicate the presence (1) or absence (0) of each virulence gene, enabling detection of different virulotypes (VTs) among the analyzed strains. Once the VTs were defined in this first analysis, 20 informative virulence genes that showed presence/absence variation across the 26 VTs were retained for subsequent multivariate analyses. Hierarchical clustering was performed using both the ClustVis server (correlation distance, complete linkage) (https://biit.cs.ut.ee/clustvis/, accessed on 12 June 2026) [42] and PAST software (version 5.2 for Mac) (Jaccard distance, UPGMA) [43]. The ClustVis server was also used to perform a Principal Component Analysis of the same VTs.

### 2.5. Strain Selection and Culture Conditions

Once the VTs of the 11 strains were identified, strains from both the ST19 and ST213 genotypes were selected for in vitro and in vivo assays under conditions that mimic GIT stress (Table 1). All the studied strains had been isolated by the State Public Health Laboratory in accordance with the federal Epidemiological Surveillance Program (NOM-017-SSA2-2012). The systematic application of this program to food samples in the state of Michoacán has not detected any ST19 strain since 2010. This is the reason why the only strain of this genotype included in the study was SAL004. Because the objective of the experimental phase was not to estimate the full phenotypic diversity of each sequence type but to compare representative virulotypes identified in the genomic survey, strains were selected to maximize virulotype representation while maintaining geographic and temporal consistency. The ATCC14028 type strain of the ST19 genotype was included in these analyses. According to ATCC, this strain was isolated from the heart and liver of a 4-week-old chicken. The *S.* Typhimurium strains, stored on French agar at room temperature, were recovered in Luria–Bertani (LB) broth and incubated at 37 °C for 18 h with orbital shaking at 130 rpm. They were then re-inoculated onto LB agar plates for experimental use.

### 2.6. GIT-Associated Conditions

#### 2.6.1. Saliva

Unstimulated whole saliva (UWS) was collected as described by Navazesh and Kumar [44]. The samples were centrifuged at 3150× *g* for 15 min to remove cellular debris, then sterilized by sequential filtration through 0.45 µm and 0.20 µm nylon membranes. The sterile saliva was stored at −80 °C and thawed gradually at 4 °C overnight before use.

For the assay, a colony of each strain was resuspended in LB broth and incubated for 18 h at 37 °C with orbital shaking at 130 rpm. From these cultures, 1 mL was used to prepare serial dilutions; 100 µL of the 1 × 10^−6^ dilution was plated on LB agar plates and spread evenly with glass beads. The plates were incubated at 37 °C for 24 h. Additionally, 3 mL of overnight culture from each strain was centrifuged at 2300× *g* for 2 min, and the supernatant was discarded. The bacterial pellets were then exposed to 1 mL of sterile UWS for 1 min as a challenge.

#### 2.6.2. Simulated Gastrointestinal Fluid

After the UWS challenge, bacterial cells were centrifuged at 2300× *g* for 2 min; the supernatant was removed, and the bacterial pellet was resuspended in simulated gastrointestinal fluid (SGF) and incubated for 2 h at 37 °C. The SGF formulation was based on Ezekiel et al. [45], with some modifications. The fluid contained an electrolyte solution with 6.2 g/L NaCl, 2.2 g/L KCl, 0.22 g/L CaCl_2_, and 1.2 g/L NaHCO_3_, to which pepsin was added at 0.3%. The pH was adjusted to 2.5.

#### 2.6.3. Simulated Intestinal Fluid

The challenged cells in SGF were centrifuged at 2300× *g* for 2 min; the supernatant was discarded, and the bacterial pellet was resuspended in simulated intestinal fluid (SIF) and incubated at 37 °C for 2 h. The composition and preparation of SIF were based on the formulation by Ezekiel et al. [45], with some modifications. SIF consists of an electrolyte solution containing 6.2 g/L of NaCl, 2.2 g/L of KCl, 0.22 g/L of CaCl_2_, and 1.2 g/L of NaHCO_3_, to which 5 mL of a 0.45% sodium deoxycholate solution and 5 mL of a 0.1% pancreatin solution were added for every 2.5 mL of the electrolyte solution. The pH was adjusted to 8.0.

At the end of the assay, bacterial cells were collected by centrifugation at 2300× *g* for 2 min, and the pellet was resuspended in phosphate-buffered saline (PBS). Then, 100 µL of each culture was spread onto LB agar plates and incubated at 37 °C for 24 h to count colony-forming units (CFU). The in vitro GIT stress survival assays were performed with eight replicates, each comprising three samples.

### 2.7. Post-Stress Recovery of the In Vitro GIT

The in vitro GIT stress protocol was performed as described in a previous section. At the end of the procedure, bacterial cells were centrifuged at 2300× *g* for 3 min, and the bacterial pellet was resuspended in 1 mL of liquid LB medium. Aliquots of 150 µL from each sample were transferred to 96-well plates and incubated at 37 °C. The optical density (OD) of the plates was measured at 595 nm using a microplate reader at 0, 2, 4, 6, 8, and 24 h after stress. The experiments were performed three times, each in triplicate.

### 2.8. Maintenance and Synchronization of C. elegans

The Bristol N2 strain of *C. elegans* was maintained on agar plates containing nematode growth medium (NGM) inoculated with 200 µL of an *Escherichia coli* OP50 culture. The bacterium was spread over three-quarters of each plate, and the plates were incubated at 30 °C overnight. Then, a 1.5 × 1.5 cm piece from a plate previously cultured with nematodes was placed in the *E. coli*-free area and incubated at 12 °C to encourage reproduction.

For synchronization, plates containing eggs and gravid hermaphrodites were selected. These plates were rinsed with M9 medium to collect worms and eggs from the agar. The liquid was transferred to a conical tube and centrifuged at 1400× *g* for 1.5 min. The supernatant was discarded, and the pellet was washed with M9 medium under the same conditions. The worms were then resuspended in a synchronization solution consisting of 1.5 mL water, 2.5 mL 1 M NaOH, and 1 mL 4% (*v*/*v*) sodium hypochlorite. The tube was agitated by hand until the worms disintegrated, as observed under a dissecting microscope. Immediately afterward, the tube was topped off with M9 medium and centrifuged again at 1400× *g* for 1.5 min; this washing step was repeated twice. Finally, the eggs were resuspended in M9 medium, transferred to plates previously inoculated with *E. coli* OP50, and incubated at 20 °C until the desired larval stage was reached.

### 2.9. Survival Tests of C. elegans in the Presence of S. Typhimurium Lawns

Two days before the worm transfer, a colony of each *S.* Typhimurium and *E. coli* OP50 strain from stock plates was suspended in 3 mL of LB medium. Cultures were incubated at 37 °C with orbital shaking at 50 rpm overnight. Then, 50 µL of each bacterial culture (1.2 × 10^6^ UFC/mL) was spread on the surface of NGM agar plates to form bacterial lawns, which were incubated at room temperature overnight. The next day, 20 N2 strain nematodes, synchronized at the L4 stage, were transferred to each plate. Survival assays were conducted at 20 °C in three independent assays, each with two replicates (six plates with 20 worms each, totaling 120 worms per bacterial strain). Survival was recorded daily, and during the oviposition period, worms were moved to fresh NGM plates with new bacterial lawns to prevent progeny loss. Nematodes that showed no movement when gently touched on the head, midsection, or tail with a platinum wire were considered dead. After all worms had died, the survival data were grouped and analyzed on the online server OASIS 2 [46].

### 2.10. C. elegans Intestine Analysis

At 48 and 96 h post-infection (PI), nematode aliquots from each treatment were collected and placed on a 2% (*w*/*v*) agarose pad on a slide for observation, then covered with a coverslip. The worms were anesthetized with 4 mM levamisole and examined under a Nikon Eclipse E800 phase-contrast microscope (Nikon Corporation, Tokyo, Japan) with a 40× objective. Measurements of the intestinal lumen diameter and the intestine were taken at 35 µm (48 h) and 55 µm (96 h) from the center of the grinder, using OMAX ToupView software, version 4.11. The total intestinal diameter was considered 100% capacity, and the percentage of the intestinal lumen occupied was calculated, which directly relates to the degree of colonization. L1-stage worms were exposed to each test strain’s lawn on NGM agar plates for 96 h at 20 °C. These measurements were performed in triplicate, with three independent replicates, and three worms were measured on each plate, so 27 worms were evaluated per strain.

### 2.11. Counting of S. Typhimurium Cells in C. elegans

To assess the growth of each *S.* Typhimurium strain in *C. elegans*, synchronized N2 nematodes were grown on an *E. coli* OP50 lawn until reaching the L4 stage. They were then washed three times—twice with M9 medium and once with 10 mM levamisole. An antibiogram profile was generated for the *S*. Typhimurium strains studied, revealing distinct antimicrobial resistance profiles. All studied strains, including *E. coli* OP50, were susceptible to ciprofloxacin at 1.5 mg/mL (see Supplementary Table S3 and associated information). Thus, to remove any externally attached bacteria, the worms were incubated for 1 h at 37 °C in a solution containing 10 mM levamisole and 1.5 mg/mL ciprofloxacin. Two additional M9 washes removed the antibiotic, and the worms were transferred to NGM plates pre-inoculated with each *S.* Typhimurium strain and incubated for 72 h at 20 °C.

For CFU counts, 10 worms from each group were collected at 24, 48, and 72 h PI. They were washed twice with 15 mM levamisole and incubated for 1 h at 37 °C in a solution of 15 mM levamisole and 1.5 mg/mL ciprofloxacin. After three washes with 15 mM levamisole, the worms were ground in a small mortar; the sediment was resuspended in 1 mL of M9, and serial dilutions were prepared. Then, 100 µL was inoculated onto *Salmonella*-*Shigella* agar plates for worms infected with *Salmonella* or onto LB agar for those from *E. coli* OP50. Plates were incubated at 37 °C for 48 h to quantify CFUs.

### 2.12. Statistical Analysis of Experimental Data

Measures of central tendency were calculated, and the Shapiro–Wilk test of normality was applied to each group. When normality was not met, nonparametric Kruskal–Wallis tests were used, followed by Dunn’s multiple-comparison test. Different letters indicate statistically significant differences between groups (*p* < 0.05), whereas groups sharing the same letter are not significantly different. All analyses were conducted using GraphPad Prism version 6.01 (GraphPad Software, Inc., San Diego, CA, USA).

The survival data for *C. elegans* were analyzed using the online server OASIS 2 [46] with the Mantel–Cox (Log-Rank) test.

For Principal Component Analysis (PCA) and Hierarchical Clustering of the experimental data, average values for each strain were calculated from experiments with *S*. Typhimurium ST19 (VTs 11 and 23) and ST213 (VTs 1 and 12). The variables included in the analyses were CFU/mL before stress, CFU/mL after stress, post-stress recovery, *C. elegans* TD_50_, % colonization 48 h PI, % colonization 96 h PI, CFU/nematode 24 h PI, CFU/nematode 48 h PI, and CFU/nematode 72 h PI. Both analyses were performed using ClustVis [42]. Prior to PCA, no data transformation was applied. Variables (rows) were mean-centered and scaled to unit variance to prevent differences in measurement scale or variance from disproportionately influencing the analysis. PCA was subsequently performed using Singular Value Decomposition (SVD) with imputation. Hierarchical clustering was performed using the correlation distance and average linkage.

## 3. Results

### 3.1. Distribution of Virulence-Related Genes in Genomes of ST19 and ST213 Strains Isolated in Mexico

A total of 119 virulence-related genes were identified across 146 analyzed genomes. Clustering based on gene presence or absence defined 26 VTs (Supplementary Table S4). The ATCC14028 strain was grouped in VT11. Eight VTs consisted solely of ST19 genomes; two consisted solely of ST213 genomes; and VT1 included genomes from both genotypes. VT1 was the most common, containing 65 ST213 genomes and 4 ST19, all with a set of 106 virulence genes. VT2, composed entirely of ST19 strains, included 22 genomes sharing 116 virulence genes. Ten genes distinguished VT1 and VT2: *gogB*, *grvA*, *pefA*, *pefB*, *pefC*, *pefD*, *rck*, *spvB*, *spvC*, and *spvR*; these were exclusive to ST19 genomes, except for *rck*, which was found in one ST213 genome from a human-origin strain isolated in 2004. The *sspH1* gene was also absent from ST213 genomes. In total, 108 virulence genes were shared between ST19 and ST213 genomes. The frequency of each gene is shown in Supplementary Table S5, highlighting 99 genes present in all 146 genomes, regardless of sample type or year of isolation. These results emphasize a conserved core of virulence-associated genes across both genotypes.

**Figure 1:**
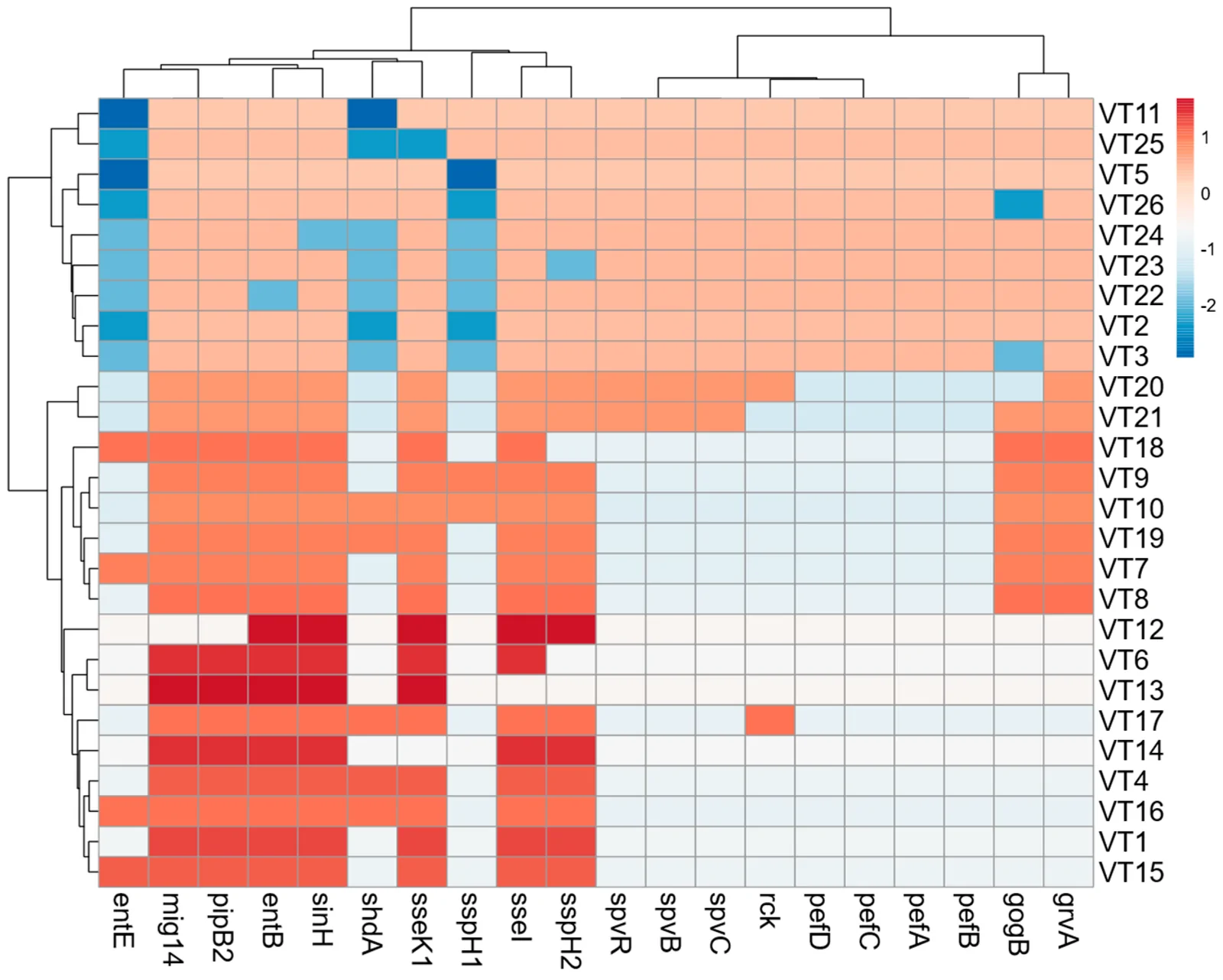
Hierarchical clustering analysis of the found *S*. Typhimurium virulotypes. Principal coordinate analysis of the binary virulence gene matrix supported the clustering patterns observed in the hierarchical clustering analysis (Supplementary Figure S1).

Using 20 informative virulence genes with variable presence across the 26 VTs, hierarchical clustering was performed. These genes include virulence plasmid-associated genes, SPI-encoded effectors, and colonization-associated determinants (Supplementary Table S6). The clustering separated the 26 VTs into four major groups (Figure 1). One group comprised virulotypes harboring the pSLT-associated genes *pefABCD*, *rck*, and *spvBCR* (VT2, VT3, VT5, VT11, VT22, VT23, VT24, VT25, VT26), whereas a second group contained virulotypes characterized by the presence of *gogB* and *grvA* (VT7, VT8, VT9, VT10, VT18, VT19). A transitional group (VT20, VT21) harbored *spvB*, *spvC*, and *spvR* genes but lacked the full *pef* repertoire. A basal four-cluster group included virulotypes that primarily possessed the conserved virulence core associated with SPI-encoded and prophage-associated determinants (VT1, VT4, VT6, VT12, VT13, VT14, VT15, VT16, VT17). Virulotypes belonging to the same ST as the strains selected for the experimental phase (VT1 and VT12 of ST213; VT11 and VT23 of ST19) clustered into distinct main groups, reflecting contrasting virulence gene repertoires. Both the Jaccard distance and the UPGMA criteria produced the same grouping pattern as observed in the ClustVis-generated heatmap.

### 3.2. In Vitro GIT Stress Survival

**Figure 2:**
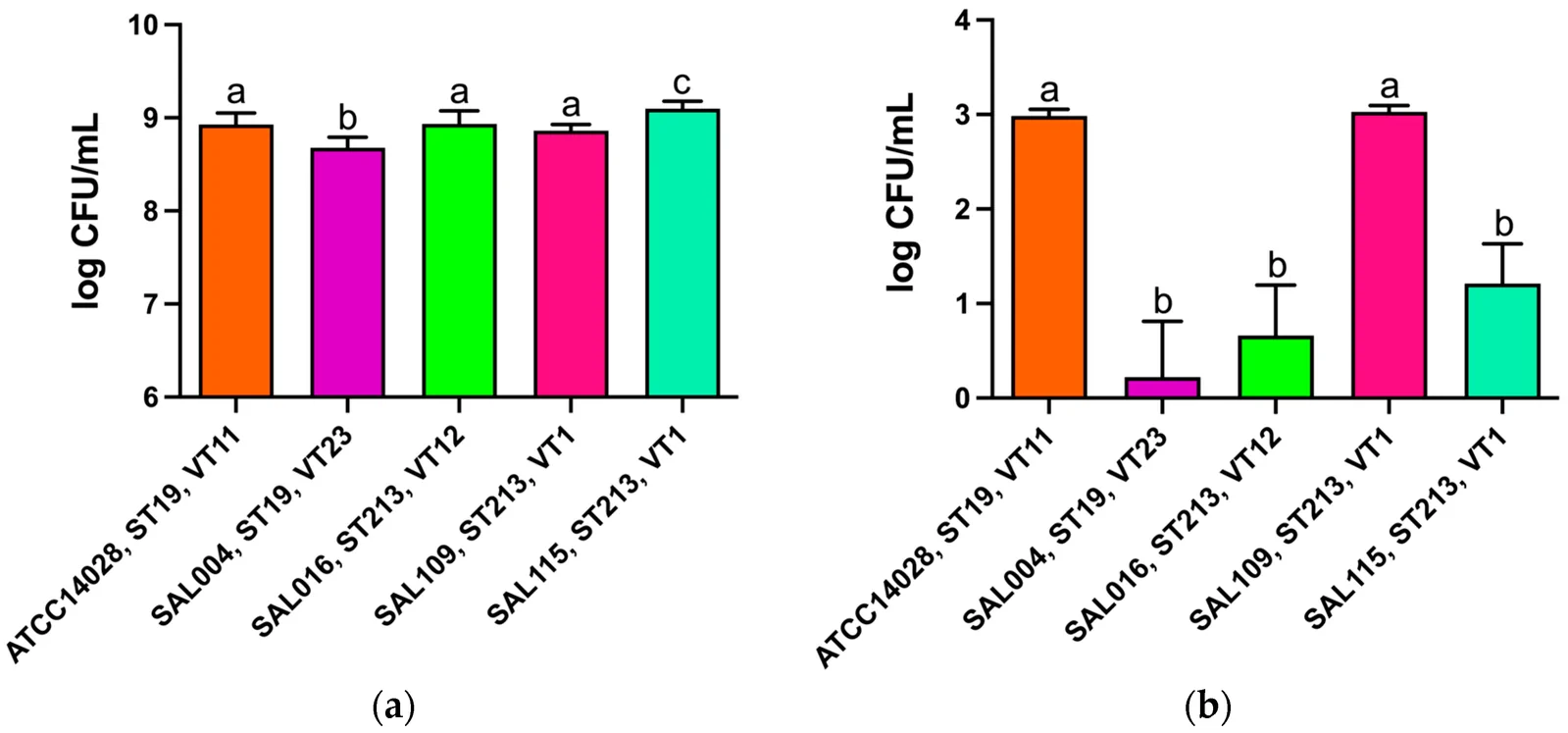
Growth of the strains analyzed before and after GIT stress: (**a**) log CFU/mL before GIT stress; (**b**) log CFU/mL after in vitro GIT stress. For both graphs, the data represent mean ± S.E. Means with different letters are significantly different (*p* < 0.05). Kruskal–Wallis with Dunn’s multiple-comparison test was used.

The analysis of CFU/mL counts before GIT stress revealed significant differences (*p* < 0.05) in growth among the selected strains based on VT. Strains ATCC 14028 (ST19, VT11), SAL016 (ST213, VT12), and SAL109 (ST213, VT1) showed similar growth. In contrast, strains SAL004 (ST19, VT23) and SAL115 (ST213, VT1) differed significantly from the previous three and from each other, with the lowest and highest CFU counts, respectively (Figure 2a). These results indicate both intragenotype and intravirulotype variation in growth before stress.

After GIT stress exposure, significant differences in survival were observed (*p* < 0.05). The representative strains ATCC14028 (ST19, VT11) and SAL109 (ST213, VT1) showed significantly higher survival rates under the simulated GIT conditions, with average survival of 967.92 and 1065.42 CFU/mL, respectively. In contrast, strains SAL004 (ST19, VT23), SAL016 (ST213, VT12), and SAL115 (ST213, VT1) exhibited similar survival levels but were significantly lower than those of ATCC14028 and SAL109. During the pre-stress phase of the GIT, differences in in vitro survival within genotypes and VTs were identified (Figure 2b).

### 3.3. S. Typhimurium Post-Stress Recovery Following In Vitro GIT Conditions

**Figure 3:**
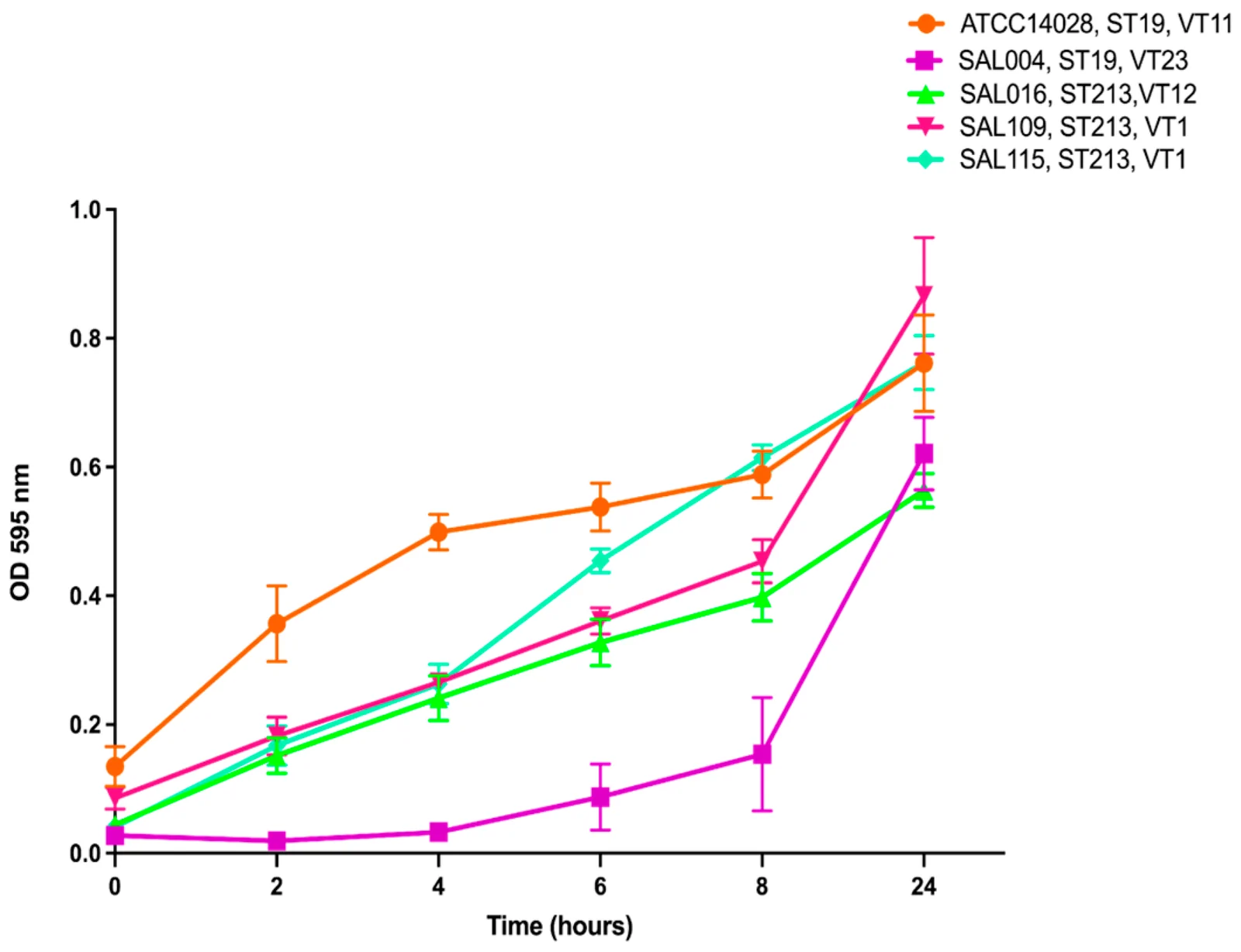
In vitro post-stress recovery of the GIT in *S.* Typhimurium strains of genotypes ST19 and ST213. Data are presented as mean ± S.E.

The recovery capacity of the strains after GIT stress was assessed (Figure 3). Consistent with the survival results, strains ATCC14028 (ST19, VT11) and SAL109 (ST213, VT1) began recovering with higher OD values than the other strains. At 2, 4, 6, and 8 h post-stress, all ST213 strains showed significantly greater recovery than SAL004 (ST19, VT23), which, even at 24 h post-stress, had a lower OD than the other ST213 strains, except for SAL016 (ST213, VT12). Although SAL115 (ST213, VT1) initially had lower recovery than SAL109 (ST213, VT1), it exhibited significantly higher OD values at 6 and 8 h post-stress. However, at 24 h, both VT1 strains, along with ATCC14028 (ST19, VT11), showed the highest recovery values among all strains (Table 2).

### 3.4. C. elegans Survival

**Figure 4:**
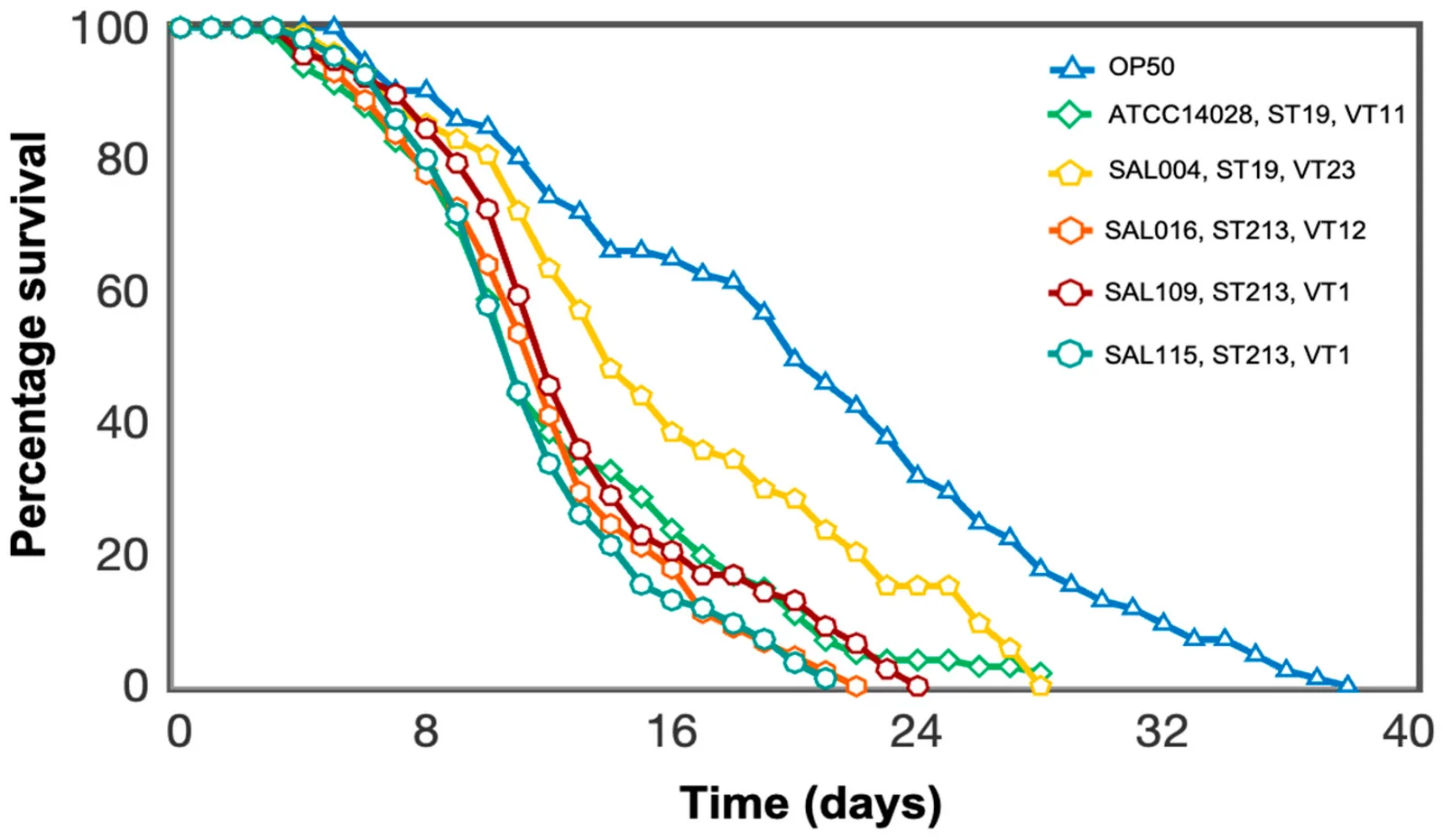
Survival of the *C. elegans* Bristol N2 strain challenged with *S.* Typhimurium strains of genotypes ST19 (VT11 and VT23) and ST213 (VT1 and VT12).

Bristol N2 strain worms at the L4 stage were exposed to lawns of *S.* Typhimurium, including ST19 (VTs 11 and 23) and ST213 (VTs 1 and 12). *E. coli* OP50 served as the control and reference for survival and median lifespan. The survival curves (Figure 4) and corresponding statistical analysis (Table 3) show that all tested *S.* Typhimurium strains significantly reduced the lifespan of *C. elegans* compared with the control, supporting their ability to reduce host survival in this infection model.

Among the evaluated strains, SAL004 (ST19, VT23) had the lowest virulence, with a time to 50% death (TD_50_) in *C. elegans* of 15.82 ± 0.73 days. The most virulent strains were SAL016 (ST213, VT12) and SAL115 (ST213, VT1), with a TD_50_ of 11.99 ± 0.4 and 11.69 ± 0.41 days, respectively. The reference strain ATCC14028 (ST19, VT11) exhibited virulence comparable to that of the other strains. These results highlight intragenotype and intravirulotype variation in the in vivo virulence of *S.* Typhimurium in *C. elegans*.

### 3.5. Colonization of the C. elegans Intestinal Tract by S. Typhimurium Genotypes

**Figure 5:**
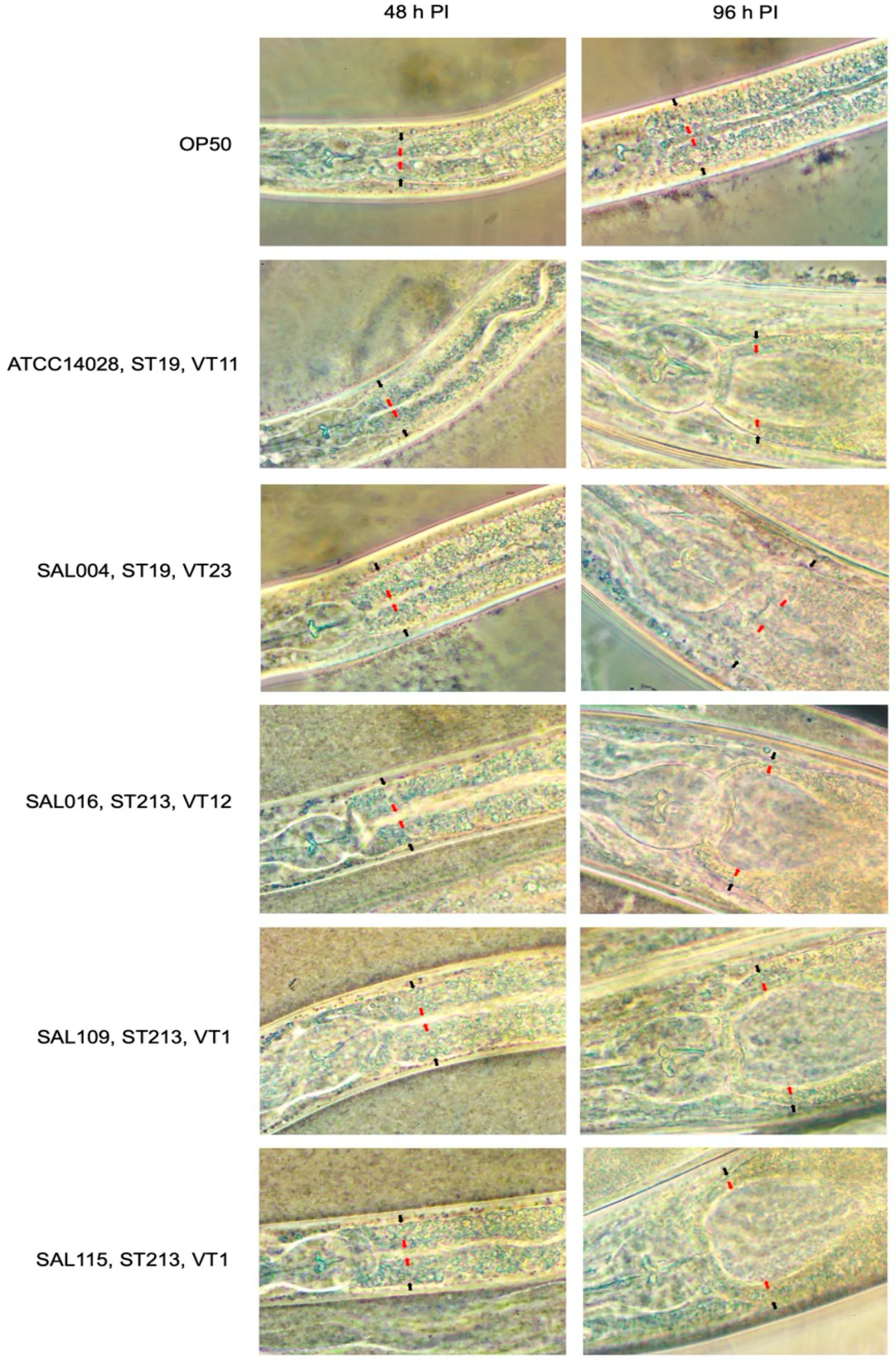
Representative micrographs of *C. elegans* at 48 and 96 h PI with *E. coli* OP50 and various strains of *S.* Typhimurium genotypes ST19 and ST213. The black arrows indicate the gut diameter, and the red arrows show the intestinal lumen. The microphotographs were taken at 40× magnification.

**Figure 6:**
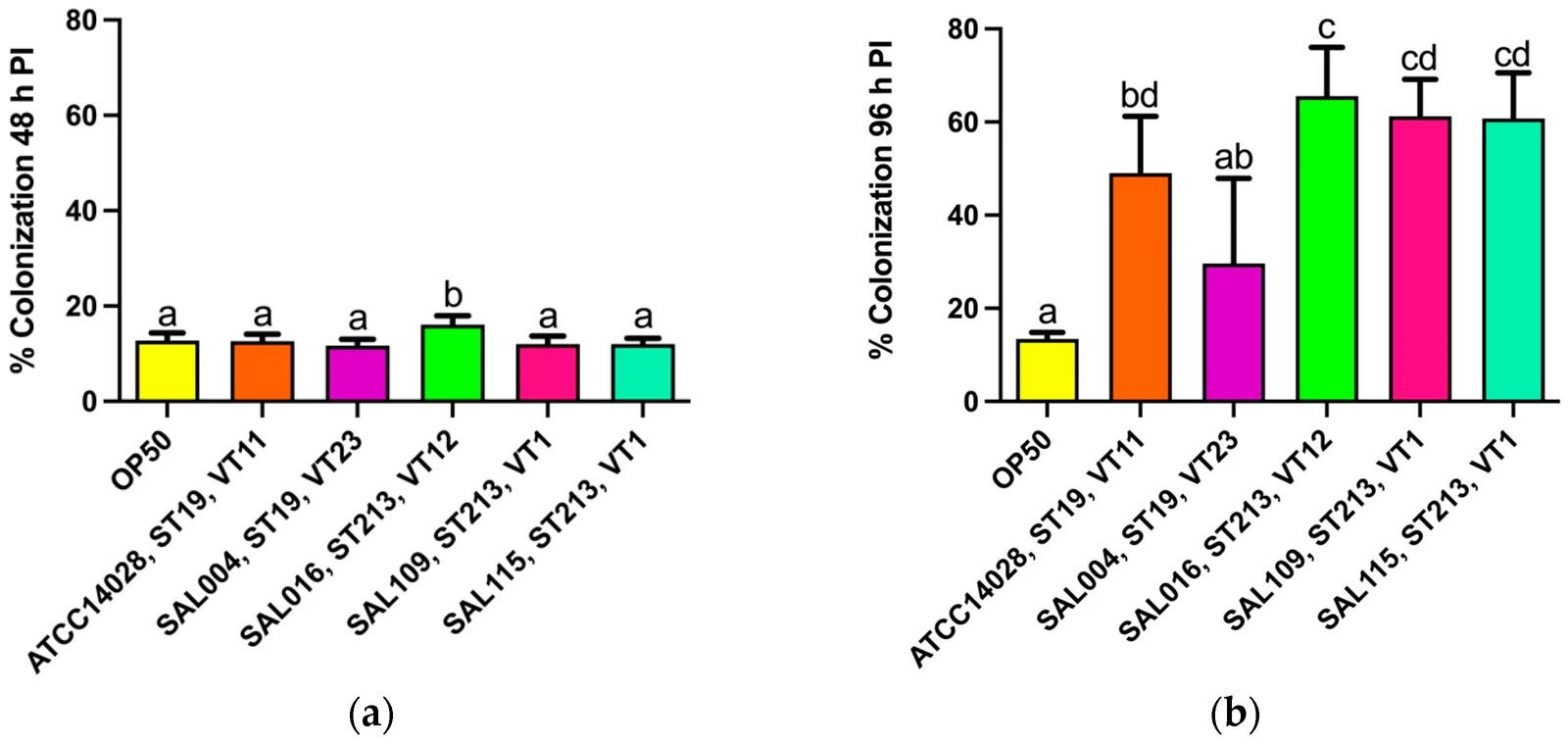
Colonization of *C. elegans* by different strains of *S.* Typhimurium ST19 (VT 11 and 23) and ST213 (VT 1 and 12). (**a**) Percentage of colonization 48 h PI in *C. elegans*. (**b**) Percentage of colonization 96 h PI in *C. elegans*. For both graphs, the data represent the mean ± S.E. Means with different letters differ significantly (*p* < 0.05). A Kruskal–Wallis test with Dunn’s multiple-comparison test was used.

L1 larvae of *C. elegans* Bristol N2 were exposed to lawns of *S.* Typhimurium with genotypes ST19, VTs 11, 23, and ST213, VTs 1, 12. The diameter of the intestinal lumen and the intestine itself were measured at 48 and 96 h PI. Both the percentage of colonization and the proportion of the lumen occupied relative to the total diameter were calculated. At 48 h PI, no intestinal lumen distension was observed in worms fed any of the strains (Figure 5); however, statistical analysis shows that worms fed strain SAL016 (ST213, VT12) have a significantly higher colonization percentage than the other groups (Figure 6a), despite the absence of observable distension. The other strains, both ST19 and ST213, show values similar to the control strain OP50, indicating no colonization at this time point.

In contrast, at 96 h PI, strains ATCC14028 (ST19, VT11), SAL016 (ST213, VT12), SAL109, and SAL115 (both ST213, VT1) caused noticeable distension of the intestinal lumen (Figure 5), which was not observed in SAL004 (ST19, VT23) or in the OP50 control. Statistical analysis of colonization percentages at 96 h PI again showed that strain SAL016 (ST213, VT12) was among the top three in colonizing the *C. elegans* intestine, followed by strains SAL109 and SAL115 (both ST213, VT1). These three strains achieved, on average, colonization percentages above 60% (Figure 6b).

Notably, despite showing no detectable colonization at 48 h PI, strains SAL109 and SAL115 caused significant distension of the intestinal lumen at 96 h PI. Conversely, the SAL004 strain (ST19, VT23) exhibited the lowest colonization percentage at 96 h PI, with values similar to those of the control *E. coli* OP50 strain, indicating minimal colonization of *C. elegans*. However, there was considerable variability among individual values, with percentages ranging from 9.42% to 64.68%. Under the conditions evaluated here, the representative ST213 strains exhibited higher *C. elegans* intestinal colonization and lumen distension than the representative ST19 strains.

### 3.6. Counting of S. Typhimurium in C. elegans

**Figure 7:**
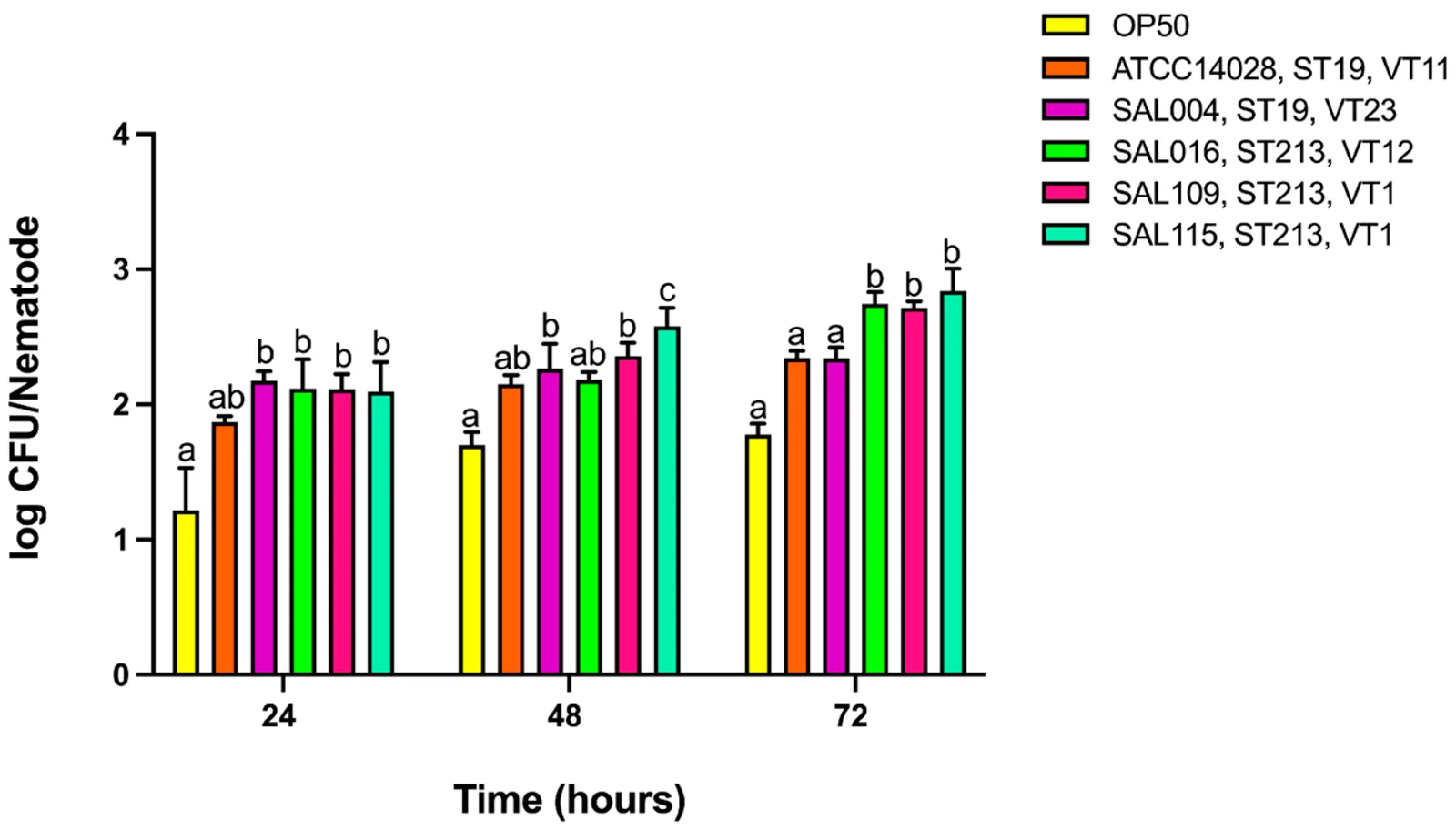
Intestinal CFU/nematode counts for each strain of *S.* Typhimurium ST19 (VT 11 and 23) and ST213 (VT 1 and 12). Data are presented as mean ± S.E. Means with different letters differ significantly between strains at each time point (*p* < 0.05). A Kruskal–Wallis test with Dunn’s multiple-comparison test was used.

Synchronized L4-stage nematodes were exposed to bacterial lawns of different *S.* Typhimurium strains, and CFUs per nematode were measured at 24, 48, and 72 h PI (Figure 7). At 24 h PI, all strains showed similar colonization levels, ranging from 74.11 CFUs/nematode (ATCC14028, ST19, VT11) to 149.22 CFUs/nematode (SAL004, ST19, VT23). Except for the ATCC14028 strain, all other values were significantly higher than those for the control strain *E. coli* OP50, which had the lowest count (16.44 CFUs/nematode).

At 48 h PI, all strains showed an increase in CFU/nematode. The control strain OP50 had the lowest count (50 CFU/nematode), with no significant difference from ATCC14028 (ST19, VT11) and SAL016 (ST213, VT12), which averaged 140.77 and 151.88 CFU/nematode, respectively. Strains SAL109 (ST213, VT1) and SAL004 (ST19, VT23) showed similar, higher counts than the previous two, while SAL115 (ST213, VT1) reached the highest value, with an average of 378.88 CFU/nematode.

Finally, at 72 h PI, both the OP50 control and all *S.* Typhimurium strains showed a further increase in CFU/nematode. The ATCC14028 (ST19, VT11) and SAL004 (ST19, VT23) strains had counts comparable to those of the OP50 strain, whereas all ST213 strains had the highest counts.

### 3.7. Clustering of S. Typhimurium Strains According to Their Physiological and Pathogenicity-Related Phenotypic Characteristics

**Figure 8:**
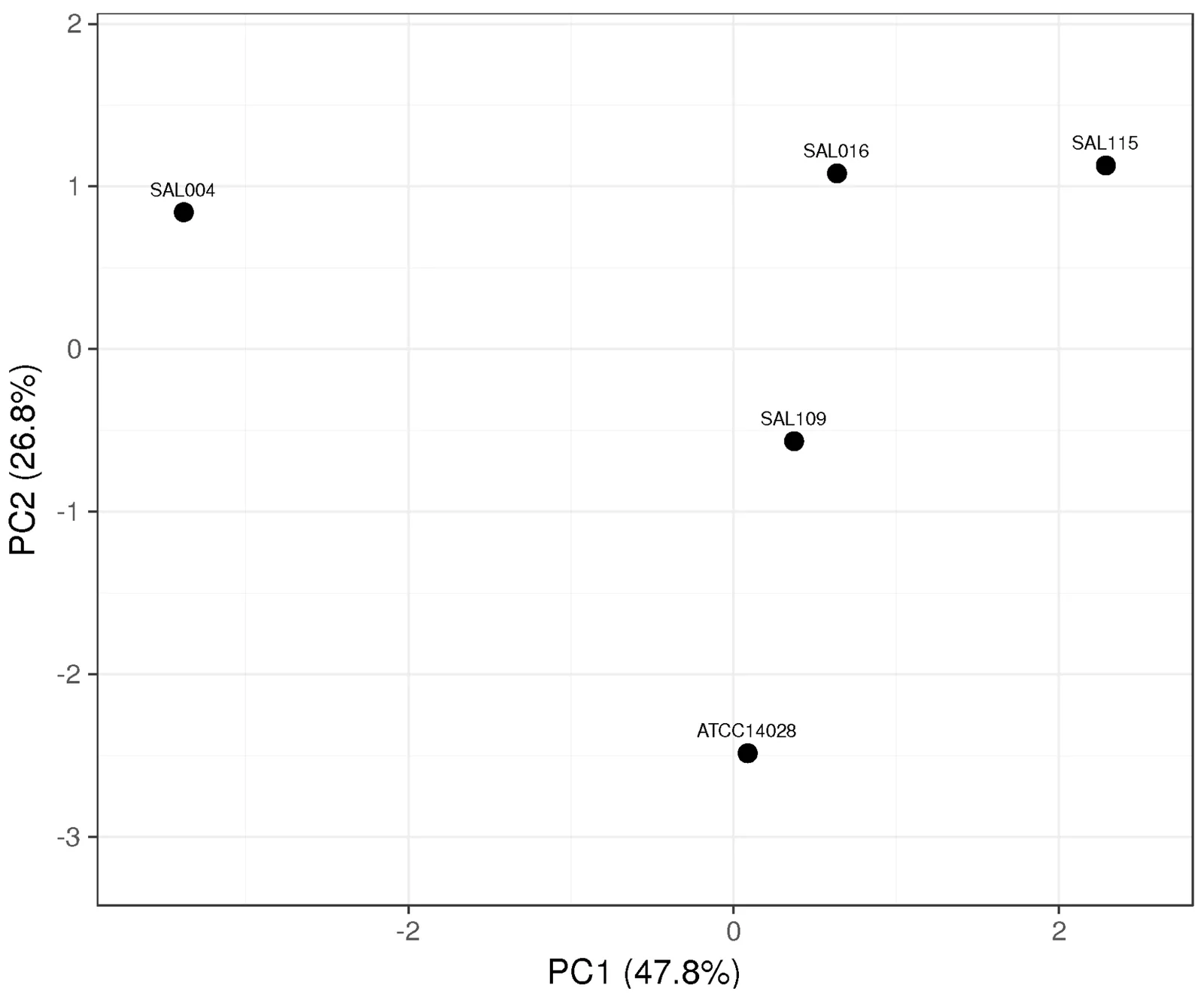
Principal Component Analysis (PCA) showing the distribution of *S*. Typhimurium strains ST19 (VT 11 and 23) and ST213 (VT 1 and 12) based on their physiological and pathogenicity-related phenotypes.

Principal component analysis (PCA) revealed differentiation among the strains based on their combined physiological and *C. elegans* infection-related phenotypes (Figure 8). The contribution of each variable (loadings) to PC1 and PC2 is presented in Supplementary Table S7. The first two principal components explained 74.6% of the total variance, with PC1 and PC2 accounting for 47.8% and 26.8%, respectively. SAL016 and SAL115 (ST213) were positioned similarly along PC2, whereas SAL109 (ST213) was distinct. SAL004 (ST19) was clearly separated from the other strains along PC1, whereas ATCC14028 (ST19) was primarily differentiated along PC2. The variables with the highest absolute loadings on PC1 were *C. elegans* TD_50_ (0.47), CFU/mL before stress (0.44), colonization at 96 h post-infection (0.43), CFU/nematode at 72 h post-infection (0.38), and post-stress recovery (0.37). PC2 was primarily associated with CFU/mL after stress (0.56), CFU/nematode at 24 h post-infection (0.55), post-stress recovery (0.38), and CFU/nematode at 72 h post-infection (0.35). Thus, strain distribution in the PCA reflected the combined contribution of stress-response traits and infection-related phenotypes rather than a single functional characteristic.

**Figure 9:**
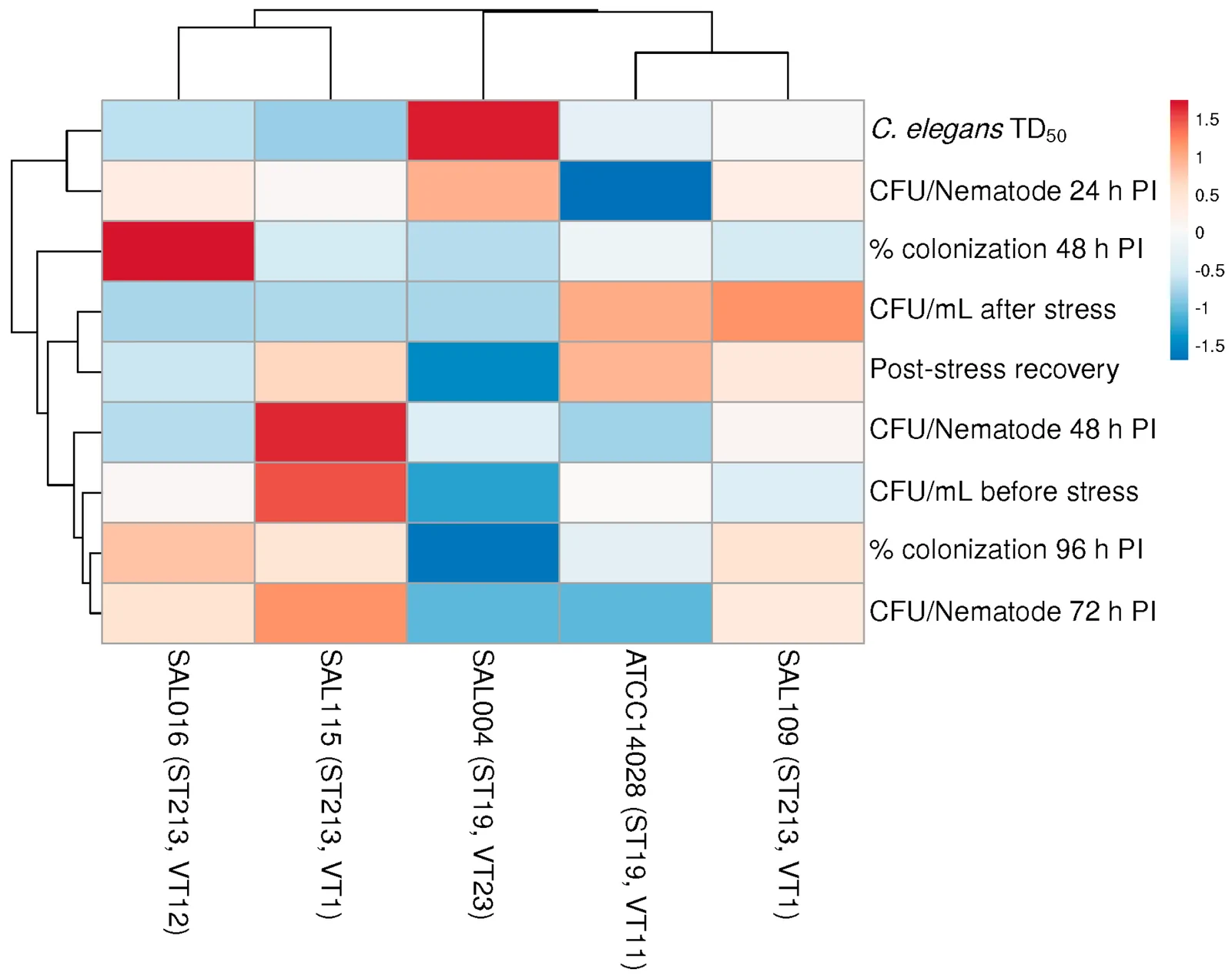
Dendrogram showing similarity and a heat map displaying the behavior of *S.* Typhimurium strains ST19 (VT 11 and 23) and ST213 (VT 1 and 12) across physiological and pathogenicity-related phenotype tests.

Hierarchical clustering of the nine standardized experimental variables grouped the strains into three major clusters (Figure 9), broadly consistent with the differentiation observed in the PCA. SAL016 (ST213, VT12) and SAL115 (ST213, VT1) formed the first cluster, sharing a similar overall phenotypic profile, particularly in infection-related traits. A second cluster comprised SAL109 (ST213, VT1) and ATCC14028 (ST19, VT11), indicating phenotypic similarity between strains belonging to different sequence types. SAL004 (ST19, VT23) formed a separate branch and displayed the most distinctive overall profile, characterized by relatively low pre-stress bacterial counts, post-stress recovery, colonization at 96 h post-infection, and bacterial burden at 72 h post-infection, together with a comparatively high TD_50_. Notably, several variables distinguishing SAL004 in the heatmap also showed high absolute loadings on PC1, supporting the consistency between the hierarchical clustering and PCA results.

## 4. Discussion

Previous studies have shown the usefulness of evaluating virulence in *C. elegans* across different *S.* Typhimurium genotypes within the same serotype and have suggested a possible relationship with specific genetic virulence factors. Analysis of five Newport serotype strains, three ST118 and one each from the ST5 and ST350 genotypes, showed that they shared 56 virulence genes, but two of the ST118 strains exhibited a chromosomal inversion and showed significant differences in the *C. elegans* virulence assay [34]. The other three strains from different STs also showed significant differences in this assay. Hoffmann et al. [33] conducted a comparative genomic analysis of 44 *S*. Heidelberg strains, mostly from the U.S., spanning the period between 1983 and 2011. Their study found a variety of prophages in the strains studied, 75 virulence-associated genes, and several plasmids. They found a variable-sized VirB/D4 plasmid in all strains, which carried elements of the type IV secretion system (T4SS), associated with conjugative translocation of DNA and effector proteins into host eukaryotic cells, as well as the uptake or dissemination of environmental DNA. To assess whether the presence of the T4SS component in this plasmid is associated with virulence, they selected six strains with different plasmid loads, finding that the three carrying the plasmid with the T4SS component significantly decreased the survival rate of *C. elegans* compared to the three that did not carry it, making it the only pathogenicity assay of the strains of interest. Thus, this work is part of a line of previous studies exploring differences in pathogenicity and virulence among strains of different serotypes/genotypes and investigating the genetic regions that may be associated with these differences.

The wide variety of virulence genes in the Mexican ST19 and ST213 strains aligns with earlier reports on the worldwide genomic structure of *S.* Typhimurium [47,48]. Previous studies have established virulotypes of *S*. Typhimurium based on the presence or absence of virulence genes. For example, 13 strains isolated from water buffalo calves with gastroenteritis were classified into 10 virulotypes, using, among others, the *spvC* and *pefA* genes to differentiate them [49]. Also, 45 strains of *S*. Typhimurium from meat of different farm animals were classified into three defined virulotypes based on the absence of the pSLT plasmid, the presence of the pSLT plasmid, and the presence of the pSLT plus the SGI-1 element [50]. A large-scale study analyzing 1081 *S*. Typhimurium genomes from databases worldwide revealed 227 distinct VTs [51]. As in the present work, the *spv* and *pef* operons within the pSLT plasmid were relevant for VT definition, being found in approximately 40% of the strains analyzed.

Kuijpers et al. [52] defined four virulence profiles in 59 strains across 32 *S. enterica* serotypes, using PCA based on the presence/absence of 101 virulence genes in their respective genomes. Profile 1 grouped the Typhimurium and Enteritidis strains, characterized by the presence of the *mig-5* and *rck* genes and *the pef* and *spv* operons, all located on the pSLT. The other three virulence profiles grouped strains belonging to the other 30 serotypes. Profile 2 grouped strains carrying the typhoid toxin genes *cdtB*, *hlyE*, *plt*, and *taiA*; Profile 3 grouped those sharing the *stk* operon virulence genes; and Profile 4 grouped those with the *peg* operon fimbrial virulence genes. Our results align with this previous work in showing that viral groups can be defined beyond the presence/absence criterion for a particular set of genes and are characterized by functional virulence modules. Thereby, the hierarchical clustering here performed shows the division of the 26 identified VTs into three main groups: one that can be called “pSLT-positive’ (characterized by the presence of the *pef*, *spv*, and *rck* genes), another that would be the “Core intracellular SPI-2′ (where the *pipB2*, *sseI*, *sseK1*, *sspH2*, and *mig-14* genes are found), and a third group, “Colonization/persistence” (which share the presence of the *shdA* and *sinH* genes). Additionally, the present work indicates that the presence of the genes/operon within the pSLT plasmid is a key factor not only for distinguishing Typhimurium from other serotypes, but also for distinguishing virulotypes within Typhimurium genotypes.

In all previously cited studies, comparisons were made between serotypes or between *S*. Typhimurium strains, without determining the genotypes of the strains analyzed. To the best of our knowledge, this is the first work to establish that different VTs can be found within the same genotype (defined as a sequence type), as is the case with ST213. Overall, the gene repertoire of the analyzed strains highlights the complex molecular machinery that enables *S.* Typhimurium genotypes to colonize hosts and survive in challenging environments.

All strains tested in this study showed a significant decrease in survival under simulated in vitro GIT stress. The most affected strain was SAL004 (ST19, VT23), with an 8-log decrease in bacterial population, whereas ATCC14028 (ST19, VT11) and SAL109 (ST213, VT1) exhibited the smallest reductions (6-log). To date, no studies have specifically examined the survival of ST213 strains under in vitro GIT stress; comparisons have been limited to the serotype level between *S*. Typhimurium and other *Salmonella* serotypes. These results are similar to those reported by Godínez-Oviedo et al. [53], who observed a 3.4-log reduction for *S.* Typhimurium ATCC14028, and fall within the range described in other studies (4.0–5.5 log CFU/mL) [54]. The larger decreases observed here may be due to differences in strain background, the composition of SGF and SIF, or the initial inoculum size.

In post-simulated in vitro GIT stress recovery assays, the ST213 strains (VT1 and VT12) recovered significantly faster than SAL004 (ST19, VT23), despite similar initial OD. This early growth advantage indicates a greater capacity for these strains to recover from gastrointestinal-like stress. Although such recovery could increase the number of viable bacteria available for subsequent host interaction, it should not be interpreted as direct evidence of enhanced colonization or pathogenicity. Research on post-simulated in vitro GIT stress recovery in *S. enterica* is limited; a previous study conducted under food-preservation conditions (4 °C, pH 3.5, and low water activity) reported similar recovery patterns for ST19 strains (ATCC14028, VT11; SAL004, VT23) and an ST213 strain (SAL115, VT1) [38]. Differences between that study and the current results may reflect methodological variations, such as the type and duration of stress applied, as well as the small number of ST213 strains previously examined.

In *S*. Typhimurium, the general stress response (RpoS), the SOS response (RecA), and the extracytoplasmic stress response (RpoE) are key systems that link stress responses both inside and outside the host, virulence, and antibiotic resistance [55]. Beyond these master regulatory systems, at least 339 *S*. Typhimurium genes associated with resistance to stresses encountered during simulated in vitro GIT passage have been identified, including those encoding the eight subunits of the FoF1-ATP synthase and genes located in pathogenicity islands, such as SPI-1 and SPI-2 [56]. Among the identified genes, *rpoS* (sigma factor), *phoP*/*phoQ*, and the *FoF1*-ATP synthase, which pumps protons to maintain internal homeostasis, participate in the Acid Tolerance Response (ATR). *rpo*E (extracytoplasmic stress), *marA*, and *invR* induce outer membrane remodeling, activate efflux pumps, and promote osmoprotectant synthesis to avoid damage from bile salts and osmotic pressure. *hilA* and *invA* (located in the SPI-1 Pathogenicity Island) help the pathogen resist damage from anaerobic environments and organic acids. Future research should determine whether these or other genes exhibit variations, such as single-nucleotide polymorphisms or other types, that help explain the differences in GIT stress responses among the studied strains.

All tested *S.* Typhimurium strains were virulent in the *C. elegans* N2 model, significantly shortening nematode lifespan compared with worms fed the control strain *E. coli* OP50. This aligns with prior reports showing 11–14-day lifespan reductions at 25 °C [57,58,59,60]. In this study, the strain that caused the greatest reduction in survival was SAL115 (ST213, VT1), which shortened survival by 11.69 ± 0.41 days, a reduction comparable to that of the highly virulent *S.* Typhimurium strain UPsm1 [61].

Although *S.* Typhimurium does not invade intestinal cells in *C. elegans*, it can form biofilms in the intestinal lumen, enabling persistent colonization even after the source of infection is removed [30,62]. Bacterial aggregates primarily accumulate in the posterior intestinal region, causing lumen distension, which is widely used as a marker of infection [63,64,65]. Similar colonization patterns have been reported in infections caused by *S.* Typhi [66] and *Enterococcus faecalis* [67]. Quantitative analysis of intestinal colonization showed a gradual increase in bacterial accumulation over time. At 72 h, the three ST213 strains maintained the highest bacterial loads, consistent with previous findings for highly virulent strains such as *S.* Typhimurium SL1344 and *S.* Typhi CT18 [66,68].

Among the VT1 virulotypes, the ST213 strains (SAL109 and SAL115) and VT12 (SAL016) show the highest colonization percentages at 96 h post-infection, whereas the VT23 strain (SAL004) exhibits the lowest colonization levels at that time point. Possible structural differences in the multidrug resistance (MDR) genetic region may contribute to variation in intestinal colonization of *C. elegans* among the *S*. Typhimurium strains studied here. Strains carrying the MDR locus are more virulent and show greater colonization and proliferation in the worm’s intestinal lumen than those that do not carry it [65]. In the future, a more detailed genomic study combined with transcriptional analysis could provide a clearer picture of the role of the MDR region in the colonization capacity of these strains. Intestinal colonization varied considerably among the strains (9.42–64.68%). This variation may reflect strain-dependent differences in bacterial ingestion, establishment, persistence, or growth within the intestinal lumen, but it may also be influenced by intrinsic biological variability among individual nematodes and experimental variation in bacterial exposure [69,70].

Although bacterial counts recovered within the first 48 h post-infection were similar across all strains, at 72 h post-infection, all ST213 strains had higher CFU/nematode counts than ST19. As expected, genes in SPI-1/SPI-2, which participate in resistance to simulated in vitro passage through the GIT, as well as pSLT, are associated with the colonization and persistence of *S*. Typhimurium in the intestine of *C. elegans* [71]. Subsequent studies showed that the *kdpD* gene, which encodes a sensor kinase of a two-component system implicated in *S*. Typhimurium pathogenesis, also contributes to the pathogen’s persistence in the worm gut [72]. Two other genetic elements that might influence intestinal colonization are the PhoP/PhoQ signal transduction system and the *fepB* gene, with the former required for virulence in *C. elegans* [30]. The latter reduces the bacterial load in the worm’s intestine, alters host immune signaling via the TGF-β pathway, and attenuates infection lethality [73]. Together, these genetic elements protect the pathogen against worm immune insults, including antimicrobial peptides and osmotic and oxidative stresses. Differences in colonization should not be interpreted as direct measures of pathogenicity, as they reflect related but independent phenotypes. Further studies will determine whether the ST213 genotype exhibits a distinct transcriptional response or harbors mutations in these genetic regions that confer greater resistance to the *C. elegans* immune response than the ST19 genotype, thereby enabling it to colonize the digestive tract.

In contrast to the present work, Cavestri et al. [74] reported that strains classified as highly virulent in murine and cellular models also exhibited increased resistance to in vivo GIT stress. The discrepancy may stem from the in vivo models used and methodological differences. The multivariate analyses conducted here indicated that phenotypic differentiation among strains was not driven by a single trait but by the combined contributions of stress-response, colonization/persistence, and host-survival phenotypes. Variables with the strongest contributions to PC1 included *C. elegans* TD_50_, bacterial population before GIT stress, colonization at 96 h PI, bacterial burden at 72 h PI, and post-stress recovery, whereas PC2 was mainly influenced by bacterial survival immediately after GIT stress and bacterial burden at 24 h PI. The hierarchical clustering was consistent with this multivariate pattern: SAL016 and SAL115 shared similar overall profiles, whereas SAL004 displayed the most distinct phenotype. Notably, SAL109 (ST213) clustered with ATCC14028 (ST19), indicating that phenotypic similarity was not strictly determined by sequence type. Together, these findings suggest that strain differentiation reflects different combinations of physiological fitness, host establishment/persistence, and effects on host survival rather than a single virulence-associated phenotype. The variability observed among strains within the same ST indicates that these phenotypic patterns cannot be attributed exclusively to sequence type and should be interpreted as strain-dependent traits.

Further analysis of ST19 strains showed that ATCC14028 (VT11) and SAL004 (VT23) exhibited distinct phenotypes. These strains contain 115 virulence genes, 103 of which are also present in VT1 and VT12. Ten genes (*gogB*, *grvA*, *pefA*, *pefB*, *pefC*, *pefD*, *rck*, *spvB*, *spvC*, and *spvR*) are unique to VT11 and VT23, while two additional genes (*mig-14* and *pipB2*) are shared with VT1. Although most of these genes are involved in adhesion, invasion, and intracellular survival, *grvA* is an anti-virulence gene whose deletion increases virulence in vivo [75]. This may partly explain the decreased infection-associated phenotypes observed in SAL004 in the *C. elegans* model. Additionally, ATCC14028 contains the genes *sspH1* and *sspH2*, which are absent in SAL004. These genes encode E3 ubiquitin ligases that interact with host signaling pathways. For example, SspH1 ubiquitinates the host kinase PKN1, thereby affecting cytoskeletal organization and immune responses [76], while SspH2 ubiquitinates host proteins such as NOD1, thereby modulating innate immune signaling [77]. The presence of these genes might explain why ATCC14028 exhibited pathogenicity-related phenotypes similar to those of ST213 strains despite sharing the same ST as SAL004.

Among ST213 strains, the only genes distinguishing VT1 from VT12 were *mig-14* and *pipB2*, present in VT1 but absent in VT12. However, SAL016 (VT12) exhibited a phenotypic profile more similar to SAL115 (VT1) than to SAL109 (VT1), despite SAL109 and SAL115 sharing the same virulotype. This indicates that virulence gene content alone is insufficient to explain the observed phenotypic variability and suggests that differential regulation of the shared virulence repertoire may contribute to these differences. At the serotype level, it has been previously documented that there is no correlation between the virulence profile, as determined by the presence or absence of virulence genes in the genomes, and resistance to in vitro GIT stress [52]. Our results showed that this lack of correlation extends to the genotype level. In *S*. Typhimurium, virulence determinants are controlled by interconnected regulatory networks that respond to environmental and host-associated signals.

Among the genetic regions whose transcriptional regulatory alterations may contribute to differences observed among the studied strains, SPI-1 and SPI-2 stand out because they play significant roles in both intraluminal colonization and other interactions with *C. elegans* that affect its longevity [68], and regulatory crosstalk also occurs between these pathogenicity islands. For example, when *S*. Typhimurium does not form a biofilm in the intestine, SptP, encoded by SPI-1, blocks the worm’s innate immune signaling mediated by p38-MAPK by reducing active SEK-1 levels, leading to faster worm death [62]. In contrast, during biofilm formation, SptP levels are low because SsrB, encoded by SPI-2, transcriptionally regulates SptP, thereby activating host defenses and favoring persistent infections. Therefore, biofilm formation is a survival strategy in prolonged infections, as increasing *C. elegans* survival benefits the parasitic lifestyle of S. Typhimurium. A recent study found no differences in nematode lifespan when comparing a strain in which the *csgD* gene, a master regulator of biofilm formation, has been deleted with a wild-type strain of *S*. Typhimurium [78]. Nevertheless, the differences between these studies may be attributable to the specific genetic region modified in each case, with the possibility of pleiotropic effects in both.

Another example of regulatory interactions among pathogenicity islands is the SPI-1 regulator HilD, which can modulate expression of the SPI-2 regulatory system, *ssrAB* [79]. Similarly, both pathogenicity islands interact with several genetic regions in the bacterial chromosome. For example, the PhoP/PhoQ system regulates *mig-14* in response to acidic pH and low Mg^2+^ and directly activates the SPI-associated effector gene *sseK1* [80,81]. The SsrA/SsrB system controls SPI-2 gene expression and effectors such as *sspH2*, while *SsrB* can counteract H-NS-mediated silencing of horizontally acquired virulence loci [82]. Thus, strains harboring highly similar virulence gene repertoires may still differ in the timing or magnitude of expression of these determinants in response to environmental or host-associated conditions. Transcriptomic or targeted gene-expression analyses under gastrointestinal stress and during host interaction will be required to determine whether such regulatory differences contribute to the phenotypic variability observed here.

These findings suggest that phenotypic variability among strains with highly conserved virulence gene repertoires may reflect differences in regulatory and metabolic adaptation [47,48] or other physiological mechanisms not captured by presence/absence analyses. Furthermore, passage of *S*. Typhimurium through the intestinal tract of *C. elegans* entails genetic changes in the SPI-1 and SPI-2 genes, including deletions, insertions, and single-nucleotide polymorphisms, which are associated with phenotypic variation, particularly in virulence [83]. Such mechanisms could influence bacterial responses to gastrointestinal stress and subsequent host interactions, although their contribution to the epidemiological success of ST213 remains to be established. Although colonization frequency, bacterial burden, and host mortality are related but distinct phenotypes, their regulation shares several genetic elements that crosstalk at multiple levels. Because only a limited number of representative strains were experimentally evaluated, the phenotypic trends identified here should be interpreted as exploratory observations rather than lineage-wide characteristics. Although the limited number of experimentally evaluated strains prevents broad generalization at the lineage level, these findings underscore the importance of integrating genomic and phenotypic approaches when comparing emerging *S*. Typhimurium lineages.

Although this study offers valuable insights, several limitations should be considered when interpreting the results. One limitation is the small number of strains included in the experimental assays, which limits the ability to generalize the observed phenotypic patterns to the broader diversity of *S.* Typhimurium across ecological or geographic environments. To minimize genotypic and phenotypic variation associated with spatiotemporal variation, this work used strains exclusively from the state of Michoacán, isolated in the same year, and for which all relevant metadata were available. The State Public Health Laboratory in Michoacán has not isolated any strains of the ST19 genotype in the last 10 years. This is significant because systematic sampling of food is conducted throughout the year and across the entire state, in accordance with the federal Epidemiological Surveillance Program (NOM-017-SSA2-2012). Based on this, ST213 and the only available ST19 strain from the past 17 years in the same region were selected for this study.

A second limitation of this work is that virulence classification was primarily based on in silico detection of virulence-associated genes, which may not reflect their expression levels or functional activity under host-related conditions. Consequently, phenotypic differences among strains with similar virulence gene profiles might arise from regulatory mechanisms that affect gene expression, post-transcriptional regulation, or metabolic adaptation. Finally, although the *C. elegans* infection model is a valuable and well-established system for studying bacterial pathogenicity and host colonization, it cannot fully replicate the complexity of mammalian host–pathogen interactions. Future research using transcriptomic analyses, regulatory network studies, and infection models in vertebrate hosts will help clarify the molecular mechanisms underlying the phenotypic variability observed among these strains.

## 5. Conclusions

The ST19 and ST213 genotypes of *S*. Typhimurium circulating in Mexico carry a largely conserved repertoire of virulence-associated genes, although differences in gene content were identified among virulotypes. Representative ST19 and ST213 strains evaluated experimentally displayed distinct phenotypic profiles in their responses to simulated GIT stress, post-stress recovery, intestinal colonization, bacterial burden, and effects on host survival in the *C. elegans* model. Under the experimental conditions evaluated, representative ST213 strains generally showed greater post-stress recovery and sustained bacterial burdens in *C. elegans*; however, considerable strain-level variability was observed, indicating that these phenotypes should not be considered lineage-wide characteristics. Differences among strains with highly similar virulence gene repertoires further suggest that gene presence alone is insufficient to explain their phenotypic behavior and that differential regulation, metabolic adaptation, or other physiological mechanisms may contribute to these strain-specific responses. Overall, these findings highlight the value of integrating genomic and phenotypic approaches to characterize the functional diversity of clinically relevant *S*. Typhimurium lineages and provide a basis for future studies examining the regulatory mechanisms underlying the observed phenotypic variability.

## Figures and Tables

**Table 1:** Information on the Mexican *S.* Typhimurium strains whose genomes were sequenced and analyzed in this study.

Strain ^1^	Code	Source	Location of Isolation	Year	ST
**SAL004**	MEXF19_2009	Cheese	Ciudad Hidalgo	2009	19
**SAL016**	MEX19F_2009	Pork	Morelia	2009	213
SAL021	MEX21F_2009	Sausage	Lázaro Cárdenas	2009	213
SAL024	MEX16F_2009	Sausage	Lázaro Cárdenas	2009	213
SAL028	MEX15F_2009	Sausage	Uruapan	2009	213
SAL039	MEX18F_2008	Beef	Apatzingán	2008	213
SAL096	MEX22F_2009	Beef	Zamora	2009	213
**SAL109**	MEX14F_2009	Beef	Zamora	2009	213
**SAL115**	MEX17F_2009	Cheese	Tanhuato	2009	213
SAL127	MEX20F_2008	Beef	La Piedad	2008	213
SAL196	MEX23F_2011	Sausage	Pátzcuaro	2011	213

Notes: ^1^ Strains in bold were selected for in vitro and in vivo tests assessing GIT stress resistance. The virulotypes (VT) of these strains are: SAL004, VT23; SAL016, VT12; SAL109, VT1; SAL115, VT1. See Supplementary Tables S1 and S2 for details.

**Table 2:** Average OD at each time point of post-stress recovery of the GIT in vitro.

	0 h	2 h	4 h	6 h	8 h	24 h
ATCC14028, ST19, VT11	0.1347 a	0.3568 a	0.4989 a	0.5380 a	0.5884 a	0.7616 a
SAL004, ST19, VT23	0.02786 c	0.01936 b	0.03291 b	0.08744 b	0.1539 b	0.6208 b
SAL016, ST213, VT12	0.04420 bc	0.1516 c	0.2411 c	0.3275 c	0.3978 c	0.5637 b
SAL109, ST213, VT1	0.08561 ab	0.1823 c	0.2658 c	0.3609 c	0.4535 c	0.8659 a
SAL115, ST213, VT1	0.04075 bc	0.1675 c	0.2633 c	0.4546 d	0.6144 a	0.7622 a

Different letters indicate statistically significant differences between strains at each time point (*p* < 0.05). ANOVA with Tukey’s multiple-comparison test was performed.

**Table 3:** TD_50_ of *C. elegans* infected with *S.* Typhimurium ST19 and ST213.

Strain	TD_50_ (Days)	Significant Difference
OP50	20.25 ± 0.92	a
ATCC14028, ST19, VT11	12.57 ± 0.55	bd
SAL004, ST19, VT23	15.82 ± 0.73	c
SAL016, ST213, VT12	11.99 ± 0.4	bd
SAL109, ST213, VT1	13.02 ± 0.47	b
SAL115, ST213, VT1	11.69 ± 0.41	d

The TD_50_ was calculated using the OASIS online server (version 2.0). Data are presented as mean ± S.E. Different letters indicate statistically significant differences (Log-Rank *p* < 0.05).

## Data Availability

The whole-genome sequencing data for all *S.* Typhimurium strains analyzed in this study have been deposited in the NCBI Sequence Read Archive (SRA) under BioProject accession number PRJNA802840. Additional public datasets utilized in this work are available under the respective SRA accession numbers listed in Supplementary Table S1.

## Supplementary Materials

The following supporting information can be downloaded at: https://www.mdpi.com/article/10.3390/microorganisms14081854/s1, Table S1: Metadata for *S*. Typhimurium strains of genotypes ST19 and ST213 isolated in Mexico, whose genomes were analyzed in this study; Table S2: Quality parameters of the genomes assembled in this work and obtained from databases for strains of genotypes ST19 and ST213 of *S.* Typhimurium isolated in Mexico; Table S3: Resistance profile of the *S*. Typhimurium strains used in *Caenorhabditis elegans* infection assays; Table S4: VTs of the ST19 and ST213 genomes analyzed in this study; Table S5: Frequency of virulence genes found in the analyzed genomes; Table S6: Data on the informative genes across virulotypes; Table S7: Contribution (loadings) of variables used in the PCA to each PC; Figure S1. Principal Coordinate Analysis of the identified VTs.

## Abbreviations

The following abbreviations are used in this manuscript:

MLST	Multilocus sequence typing
WGCA	Whole-genome comparative analysis
ST	Sequence type
VT	Virulotype
VTs	Virulotypes
LESPM	Michoacán state public health laboratory
GIT	Gastrointestinal tract
LB	Luria–Bertani
UWS	Unstimulated whole saliva
SGF	Simulated gastrointestinal fluid
SIF	Simulated intestinal fluid
PBS	Phosphate-buffered saline
CFU	Colony-forming units
OD	Optical density
NGM	Nematode growth medium
PI	Post-infection
SCVs	*Salmonella*-containing vacuoles
