# Supplementary material for: Similar Virulence Gene Repertoires but Distinct Stress Tolerance and Pathogenicity-Associated Phenotypes in Representative Salmonella Typhimurium ST19 and ST213 Strains from Mexico
Source: Microorganisms. 2026 Aug 20;14(8):1854. doi: 10.3390/microorganisms14081854 (PMC13515107; doi:10.3390/microorganisms14081854)
Supplement: Supplementary file 1 [file microorganisms-14-01854-s001.zip › microorganisms-4481037-supplementary.pdf]

Supplementary Table S1. Metadata for *S.* Typhimurium strains of genotypes ST19 and ST213 isolated in Mexico, whose genomes were analyzed in this study.

Code	Access number	Source	Year	ST
MEXF01_2010	SRR1177271	Food	2010	19
MEXF02_2011	SRR1427103	Food	2011	19
MEXB03_2018	SRR10189752	Bovine	2018	19
MEXB04_2018	SRR10189753	Bovine	2018	19
MEXB05_2018	SRR10189757	Bovine	2018	19
MEXB06_2018	SRR10189766	Bovine	2018	19
MEXB07_2018	SRR10189767	Bovine	2018	19
MEXB08_2018	SRR10189768	Bovine	2018	19
MEXEq09_2018	SRR9652069	Equine	2018	19
MEXEq10_2018	SRR9652071	Equine	2018	19
MEXEq11_2018	SRR9652084	Equine	2018	19
MEXF12_2010	SRR1035566	Food	2010	19
MEXF13_2021	SRR14943705	Food	2021	19
MEXF14_2011	SRR1593423	Food	2011	19
MEXF15_2009	SRR1685381	Food	2009	19
MEXF16_2004	SRR2104619	Food	2004	19
MEXF17_2002	SRR2878365	Food	2002	19
MEXF19_2009	SRR18072959	Food	2009	19
MEXF18_2003	SRR4427077	Food	2003	19
MEXF21_2011	traces-0kWgwJD*	Food	2011	19
MEXH22_2004	ERR2693956	Human	2004	19
MEXH23_2000	ERR2693960	Human	2000	19
MEXH24_2001	ERR2719666	Human	2001	19
MEXH25_2002	ERR2719675	Human	2002	19
MEXH26_2000	ERR2719686	Human	2000	19
MEXH27_2004	ERR2719794	Human	2004	19
MEXH28_2004	ERR2719913	Human	2004	19
MEXH29_2002	ERR4401231	Human	2002	19
MEXH30_2003	ERR4413432	Human	2003	19
MEXH31_2001	ERR2693683	Human	2001	19
MEXH32_2004	ERR2710831	Human	2004	19
MEXH33_2000	ERR2710842	Human	2000	19
MEXH34_2003	ERR2710857	Human	2003	19
MEXH35_2002	ERR2710866	Human	2002	19
MEXH36_2002	ERR2710886	Human	2002	19
MEXH37_2000	ERR2719650	Human	2000	19
MEXH38_2004	ERR3901517	Human	2004	19

MEXH39_2003	ERR3901359	Human	2003	19
MEXH40_2001	ERR3901483	Human	2001	19
MEXH41_2000	GCF_001576255	Human	2000	19
MEXN42_1969	traces-0zaCRhK*	Other	1969	19
MEXS43_2002	ERR4413367	Pork	2002	19
MEXS44_2004	ERR2710826	Pork	2004	19
MEXR45_2018	traces-0JkyULj	Water/river	2018	19
MEXWa46_2008	SRR3092104	Wild animal	2008	19
MEXWa47_2008	SRR3092106	Wild animal	2008	19
MEXH48_2004	ERR2693595	Human	2004	19
MEXH49_2003	ERR2693640	Human	2003	19
MEXH50_2003	ERR2719863	Human	2003	19
MEXR51_2021	SRR21535520	Water/river	2021	19
MEXR52_2021	SRR21535509	Water/river	2021	19
MEXR53_2021	SRR21536562	Water/river	2021	19
MEXF54_2004	SRR9017231	Food	2004	19
MEXB55_2017	SRR9831928	Bovine	2017	19
MEXR56_2018	traces-0iyLSen*	Water/river	2018	19
MEXR57_2021	SRR21536563	Water/river	2021	19
MEXSD58_2021	SRR21536526	Soil/dust	2021	19
MEXR59_2021	SRR21787367	Water/river	2021	19
MEXR60_2021	SRR21787279	Water/river	2021	19
MEXR61_2022	SRR22230402	Water/river	2022	19
MEXR62_2019	SRR22230686	Water/river	2019	19
MEXR63_2021	SRR22231360	Water/river	2021	19
MEXR64_2019	SRR22234162	Water/river	2019	19
MEX14F_2009	SRR23312944	Food	2009	213
MEX15F_2009	SRR23312947	Food	2009	213
MEX16F_2009	SRR23312948	Food	2009	213
MEX17F_2009	SRR23312943	Food	2009	213
MEX18F_2008	SRR23312946	Food	2008	213
MEX19F_2009	SRR23312950	Food	2009	213
MEX20F_2008	SRR23312942	Food	2008	213
MEX21F_2009	SRR23312949	Food	2009	213
MEX22F_2009	SRR23312945	Food	2009	213
MEX23F_2011	SRR23312941	Food	2011	213
MEX68R_2020	SRR18517463	Water/river	2020	213
MEX69R_2021	SRR18544685	Water/river	2021	213
MEX70R_2021	SRR18544710	Water/river	2021	213
MEX71R_2021	SRR18544717	Water/river	2021	213
MEX72R_2021	SRR18544718	Water/river	2021	213

MEX73B_2021	SRR20081726	Bovine	2021	213
MEX74R_2021	SRR21786912	Water/river	2021	213
MEX75R_2021	SRR21786918	Water/river	2021	213
MEX76R_2019	SRR22230420	Water/river	2019	213
MEX77R_2019	SRR22230367	Water/river	2019	213
MEX78R_2020	SRR22230231	Water/river	2020	213
MEX79R_2019	SRR22229564	Water/river	2019	213
MEX80R_2020	SRR22228159	Water/river	2020	213
MEX81R_2020	SRR22228155	Water/river	2020	213
MEX82R_2020	SRR22228152	Water/river	2020	213
MEX83R_2020	SRR22234204	Water/river	2020	213

Notes: ST, Sequence Type. *Genomes obtained from the Enterobase database (https://enterobase.warwick.ac.uk/) using internal trace accession codes. These assemblies are curated within Enterobase but are not available in standard public repositories.

Supplementary Table S2. Quality parameters of the genomes assembled in this work and obtained from databases for strains of genotypes ST19 and ST213 of *S.* Typhimurium isolated in Mexico.

Code	Access No.	ST	No. Contigs	Genome length	Largest contig	Average contig length	N_{50}	GC
MEXF01_2010	SRR1177271	19	78	4933168	656567	8417.53	324913	52.2
MEXF02_2011	SRR1427103	19	76	4957364	694200	9134.21	376787	52.15
MEXB03_2018	SRR10189752	19	127	4934131	454406	3578.00	172301	52.13
MEXB04_2018	SRR10189753	19	214	4956805	322795	1508.39	109289	52.13
MEXB05_2018	SRR10189757	19	103	4934210	545986	5300.83	166596	52.12
MEXB06_2018	SRR10189766	19	118	4940753	382087	3238.03	172302	52.12
MEXB07_2018	SRR10189767	19	103	4939111	454740	4414.95	175428	52.12
MEXB08_2018	SRR10189768	19	117	4935917	441745	3775.60	181079	52.13
MEXEQ09_2018	SRR9652069	19	87	4915822	820846	9435.01	276502	52.15
MEXEQ10_2018	SRR9652071	19	108	4928504	929738	8608.69	324780	52.14
MEXEQ11_2018	SRR9652084	19	99	4917152	929738	9391.29	222891	52.15
MEXF12_2010	SRR1035566	19	87	4944954	548627	6306.06	325559	52.18
MEXF13_2021	SRR14943705	19	84	4914954	660529	7863.44	302871	52.19
MEXF14_2011	SRR1593423	19	99	4859719	626937	6332.70	271004	52.21

MEXF15_2009	SRR1685381	19	214	4980199	590974	2761.56	219123	52.09
MEXF16_2004	SRR2104619	19	81	4931112	656451	8104.33	324913	52.2
MEXF17_2002	SRR2878365	19	186	5024293	274961	1478.28	76343	52.18
MEXF18_2003	SRR4427077	19	66	4884523	571229	8654.98	325073	52.21
MEXF19_2009	SRR18072959	19	41	4835679	660690	16114.39	377051	52.21
MEXF21_2011	traces-0kWgwJD*	19	69	5036984	590791	8562.19	271384	52.18
MEXH22_2004	ERR2693956	19	244	5031524	471846	1933.80	195939	52.12
MEXH23_2000	ERR2693960	19	206	5047674	546726	2654.01	204550	52.1
MEXH24_2001	ERR2719666	19	201	4886378	394193	1961.16	159060	52.11
MEXH25_2002	ERR2719675	19	167	4941361	577663	3459.06	341535	52.17
MEXH26_2000	ERR2719686	19	213	4929062	306158	1437.36	163592	52.08
MEXH27_2004	ERR2719794	19	226	4850489	527667	2334.81	191582	52.16
MEXH28_2004	ERR2719913	19	164	4947838	576825	3517.23	342054	52.13
MEXH29_2002	ERR4401231	19	146	5036698	524381	3591.65	341536	52.19
MEXH30_2003	ERR4413432	19	185	4957518	443686	2398.30	193791	52.18
MEXH31_2001	ERR2693683	19	56	5094706	606156	10824.21	222567	52.08
MEXH32_2004	ERR2710831	19	58	4944233	513255	8849.22	207445	52.07
MEXH33_2000	ERR2710842	19	91	5090559	318179	3496.47	149672	51.88
MEXH34_2003	ERR2710857	19	56	4995461	608341	10863.23	204859	52.1
MEXH35_2002	ERR2710866	19	64	5111854	538415	8412.73	223874	52.16
MEXH36_2002	ERR2710886	19	54	4962343	682005	12629.72	204859	52.11
MEXH37_2000	ERR2719650	19	78	5079872	302051	3872.45	147369	51.86
MEXH38_2004	ERR3901517	19	46	4760640	527300	11463.04	207445	52.18
MEXH39_2003	ERR3901359	19	47	4966805	523652	11141.53	196158	52.11
MEXH40_2001	ERR3901483	19	65	4850243	407102	6263.11	139590	52.13
MEXH41_2000	GCF_001576255	19	2	5027649	4933574	2466787.00	4933574	52.13
MEXN42_1969	traces-0zaCRhK*	19	57	4923103	908212	15933.54	301494	52.17
MEXS43_2002	ERR4413367	19	202	5016542	522876	2588.50	196263	52.1
MEXS44_2004	ERR2710826	19	57	5073510	717608	12589.61	215426	51.74
MEXR45_2018	traces-0JkyULj*	19	111	4890406	622466	5607.80	121322	52.16
MEXWA46_2008	SRR3092104	19	85	4889321	471833	5550.98	199316	52.19
MEXWA47_2008	SRR3092106	19	86	4969488	627321	7294.43	223715	52.16

MEXH48_2004	ERR2693595	19	60	4941669	682279	11371.32	225813	52.12
MEXH49_2003	ERR2693640	19	82	4999515	746462	9103.20	257733	52.1
MEXH50_2003	ERR2719863	19	59	4917826	627390	10633.73	271390	52.16
MEXR51_2021	SRR21535520	19	57	4753510	720014	12631.82	322100	52.15
MEXR52_2021	SRR21535509	19	55	4752542	720014	13091.16	322100	52.15
MEXR53_2021	SRR21536562	19	100	4807115	542984	5429.84	267545	52.16
MEXF54_2004	SRR9017231	19	96	5094087	715658	7454.77	210718	51.76
MEXB55_2017	SRR9831928	19	127	4879876	276251	2175.20	99079	52.2
MEXR56_2018	traces-0iyLSen*	19	107	4889615	718779	6717.56	118349	52.16
MEXR57_2021	SRR21536563	19	65	4812555	577010	8877.08	376793	52.16
MEXSD58_2021	SRR21536526	19	115	4796848	542907	4720.93	253214	52.16
MEXR59_2021	SRR21787367	19	75	4813915	542846	7237.95	376793	52.16
MEXR60_2021	SRR21787279	19	54	4752483	720014	13333.59	282798	52.15
MEXR61_2022	SRR22230402	19	66	4905277	603095	9137.80	376792	52.2
MEXR62_2019	SRR22230686	19	68	4978946	731339	10754.99	225812	52.1
MEXR63_2021	SRR22231360	19	67	4907509	547045	8164.85	461145	52.19
MEXR64_2019	SRR22234162	19	68	4848066	545716	8025.24	290013	52.21
MEX14F_2009	PRJNA802840	213	69	4845819	621554	9008.03	175184	52.08
MEX15F_2009	SRR23312944	213	36	4799962	714664	19851.78	301237	52.15
MEX16F_2009	SRR23312947	213	64	5132286	881896	13779.63	259009	51.87
MEX17F_2009	SRR23312948	213	34	4924086	881344	25921.88	301228	52.03
MEX18F_2008	SRR23312943	213	51	4961687	700153	13728.49	283075	52.1
MEX19F_2009	SRR23312946	213	48	4991223	690570	14386.88	282543	52.09
MEX20F_2008	SRR23312950	213	38	4965399	872543	22961.66	298066	52.05
MEX21F_2009	SRR23312942	213	50	5020395	875226	17504.52	282876	52.09
MEX22F_2009	SRR23312949	213	44	4877413	786859	17883.16	283075	51.97
MEX23F_2011	SRR23312945	213	126	5033426	309052	2452.79	102488	52.06
MEX68R_2020	SRR18517463	213	55	5060198	497657	9048.31	282543	51.85
MEX69R_2021	SRR18544685	213	40	4936825	819130	20478.25	376541	52.02
MEX70R_2021	SRR18544710	213	39	4815462	778337	19957.36	321224	52.12
MEX71R_2021	SRR18544717	213	50	5008651	881346	17626.92	376541	52.12
MEX72R_2021	SRR18544718	213	39	4815013	778521	19962.08	301228	52.12

MEX73B_2021	SRR20081726	213	65	5093678	497917	7660.26	282543	51.91
MEX74R_2021	SRR21786912	213	57	4982175	964068	16913.47	301490	52.04
MEX75R_2021	SRR21786918	213	59	4982849	819457	13889.10	364216	52.04
MEX76R_2019	SRR22230420	213	55	4841194	888701	16158.20	376801	52.13
MEX77R_2019	SRR22230367	213	56	4841530	1054616	18832.43	394003	52.13
MEX78R_2020	SRR22230231	213	61	4911619	888517	14565.85	265101	52.11
MEX79R_2019	SRR22229564	213	55	4952176	881057	16019.22	324878	52.02
MEX80R_2020	SRR22228159	213	48	4851796	881828	18371.42	320588	52.11
MEX81R_2020	SRR22228155	213	50	4883953	779023	15580.46	272910	52.09
MEX82R_2020	SRR22228152	213	54	4886664	881261	16319.65	301484	52.09
MEX83R_2020	SRR22234204	213	63	4853279	667294	10591.97	241598	52.11

Notes: Avg. Contigs length, Average contig length. *Genomes obtained from the Enterobase database (https://enterobase.warwick.ac.uk/) using internal trace accession codes. These assemblies are curated within Enterobase but are not available in standard public repositories.

Supplementary Table S3. Resistance profile of the *S*. Typhimurium strains used in *Caenorhabditis elegans* infection assays.

Strain/ST/VT	Resistance profile
ATCC14028/ST19/VT11	S*
SAL004/ST19/VT23	S
SAL016/ST213/VT12	cefalexin, gentamicin, trimethoprim-sulfamethoxazole
SAL109/ST213/VT1	ampicillin, carbenicillin, cephalothin, cefotaxime, ceftriaxone, cefalexin, gentamicin, netilmicin
SAL115/ST213/VT1	ampicillin, carbenicillin, cephalothin, cefalexin
Escherichia coli OP50	S

* Note: S indicates susceptibility to all antibiotics tested at all concentrations used.

Because SAL016, SAL109, and SAL115 were multidrug-resistant at the antibiotic concentrations used in the Kirby-Bauer assay (Table S3), higher concentrations of various antibiotics were tested to determine which antibiotic—and at what concentration—was effective against all three. Susceptibility testing was performed by exposing the strains to antibiotics and evaluating growth, in accordance with the recommendations and standards of the Clinical and Laboratory Standards Institute (CLSI). The antibiotics tested were ceftazidime (1 mg/mL), amikacin (0.1 mg/mL), and levofloxacin, with ciprofloxacin tested at 0.1, 1, 1.5, and 2 mg/mL. Each strain was cultured on Luria-Bertani (LB) agar plates and incubated for 24 hours at 37°C. A bacterial suspension equivalent to 0.5 on the McFarland scale (OD600 = 0.08–0.1) was then prepared. Aliquots of the bacterial suspension and the antibiotic solutions were placed in 1.5 mL tubes and incubated for 1 hour at 37°C. After this period, 100 μL aliquots from each treatment were spread onto LB agar plates using the surface extension technique. Plates were incubated for 24 hours at 37°C, and bacterial growth was evaluated

as an indicator of antibiotic susceptibility. No colony-forming units were observed for any strain on plates containing ciprofloxacin at 1.5 mg/mL; this concentration was used to incubate the worms to eliminate bacteria attached to the body surface.

Supplementary Table S4. VTs of the ST19 and ST213 genomes analyzed in this study.

VT	Genomes	Genes[1]	ST	Number of genes
VT1	4<br>65	*entB*, *mig-14*, *pipB2*, *sinH*, *sseI/srfH*, *sseK1* and *sspH2*.	19<br>213	106
VT2	22	*entB*, *gogB*, *grvA*, *mig-14*, *pefA*, *pefB*, *pefC*, *pefD*, *pipB2*, *rck*, *sinH*, *spvB*, *spvC*, *spvR*, *sseI/srfH*, *sseK1* and *sspH2*.	19	116
VT3	8	*entB*, *grvA*, *mig-14*, *pefA*, *pefB*, *pefC*, *pefD*, *pipB2*, *rck*, *sinH*, *spvB*, *spvC*, *spvR*, *sseI/srfH*, *sseK1* and *sspH2*.	19	115
VT4	8	*entB*, *mig-14*, *pipB2*, *shdA*, *sinH*, *sseI/srfH*, *sseK1* and *sspH2*.	213	107
VT5	7	*entB*, *gogB*, *grvA*, *mig-14*, *pefA*, *pefB*, *pefC*, *pefD*, *pipB2*, *rck*, *shdA*, *sinH*, *spvB*, *spvC*, *spvR*, *sseI/srfH*, *sseK1* and *sspH2*.	19	117
VT6	5	*entB*, *mig-14*, *pipB2*, *sinH*, *sseI/srfH* and *sseK1*.	213	105
VT7	4	*entB*, *entE*, *gogB*, *grvA*, *mig-14*, *pipB2*, *sinH*, *sseI/srfH*, *sseK1* and *sspH2*.	19	109
VT8	2	*entB*, *gogB*, *grvA*, *mig-14*, *pipB2*, *sinH*, *sseI/srfH*, *sseK1* and *sspH2*.	19	108
VT9	2	*entB*, *gogB*, *grvA*, *mig-14*, *pipB2*, *sinH*, *sseI/srfH*, *sseK1*, *sspH1* and *sspH2*.	19	109
VT10	2	*entB*, *gogB*, *grvA*, *mig-14*, *pipB2*, *shdA*, *sinH*, *sseI/srfH*, *sseK1*, *sspH1* and *sspH2*.	19	110
VT11	2	*entB*, *gogB*, *grvA*, *mig-14*, *pefA*, *pefB*, *pefC*, *pefD*, *pipB2*, *rck*, *sinH*, *spvB*, *spvC*, *spvR*, *sseI/srfH*, *sseK1*, *sspH1* and *sspH2*.	19	117
VT12	1	*entB*, *sinH*, *sseI/srfH*, *sseK1* and *sspH2*.	213	104
VT13	1	*entB*, *mig-14*, *pipB2*, *sinH* and *sseK1*.	213	104
VT14	1	*entB*, *mig-14*, *pipB2*, *sinH*, *sseI/srfH*, and *sspH2*.	19	105
VT15	1	*entB*, *entE*, *mig-14*, *pipB2*, *sinH*, *sseI/srfH*, *sseK1* and *sspH2*.	213	107
VT16	1	*entB*, *entE*, *mig-14*, *pipB2*, *shdA*, *sinH*, *sseI/srfH*, *sseK1* and *sspH2*.	213	108
VT17	1	*entB*, *mig-14*, *pipB2*, *rck*, *shdA*, *sinH*, *sseI/srfH*, *sseK1* and *sspH2*.	213	108
VT18	1	*entB*, *entE*, *gogB*, *grvA*, *mig-14*, *pipB2*, *sinH*, *sseI/srfH* and *sseK1*.	19	108
VT19	1	*entB*, *gogB*, *grvA*, *mig-14*, *pipB2*, *shdA*, *sinH*, *sseI/srfH*, *sseK1* and *sspH2*.	19	109
VT20	1	*entB*, *grvA*, *mig-14*, *pipB2*, *rck*, *sinH*, *spvB*, *spvC*, *spvR*, *sseI/srfH*, *sseK1* and *sspH2*.	19	111
VT21	1	*entB*, *gogB*, *grvA*, *mig-14*, *pipB2*, *sinH*, *spvB*, *spvC*, *spvR*, *sseI/srfH*, *sseK1* and *sspH2*.	19	111

VT22	1	*gogB, grvA, mig-14, pefA, pefB, pefC, pefD, pipB2, rck, sinH, spvB, spvC, spvR, sseI/srfH, sseK1* and *sspH2.*	19	115
VT23	1	*entB, gogB, grvA, mig-14, pefA, pefB, pefC, pefD, pipB2, rck, sinH, spvB, spvC, spvR, sseI/srfH* and *sseK1.*	19	115
VT24	1	*entB, gogB, grvA, mig-14, pefA, pefB, pefC, pefD, pipB2, rck, spvB, spvC, spvR, sseI/srfH, sseK1* and *sspH2.*	19	115
VT25	1	*entB, gogB, grvA, mig-14, pefA, pefB, pefC, pefD, pipB2, rck, sinH, spvB, spvC, spvR, sseI/srfH, sspH1* and *sspH2.*	19	116
VT26	1	*entB, grvA, mig-14, pefA, pefB, pefC, pefD, pipB2, rck, shdA, sinH, spvB, spvC, spvR, sseI/srfH, sseK1* and *sspH2.*	19	116

Notes: [1] The remaining 99 conserved virulence-associated genes 99 present in 100% of genomes are listed in Supplementary Table S4.

Supplementary Table S5. Frequency of virulence genes found in the analyzed genomes.

Genes	Frequency (%)
avrA, csgA, csgB, csgC, csgD, csgE, csgF, csgG, entA, fepC, fepG, fimC, fimD, fimF, fimH, fimI, invA, invB, invC, invE, invF, invG, invH, invI, invJ, lpfA, lpfB, lpfC, lpfD, lpfE, mgtB, mgtC, misL, ompA, orgA, orgB, orgC, pipB, prgH, prgI, prgJ, prgK, ratB, sicA, sicP, sifA, sifB, sipA/sspA, sipB/sspB, sipC/sspC, sipD, slrP, sodCI, sopA, sopB/sigD, sopD, sopD2, sopE2, spaO, spaP, spaQ, spaR, spaS, spiC/ssaB, sptP, ssaC, ssaD, ssaE, ssaG, ssaH, ssaI, ssaJ, ssaK, ssaL, ssaM, ssaN, ssaO, ssaP, ssaQ, ssaR, ssaS, ssaT, ssaU, ssaV, sscA, sscB, sseA, sseB, sseC, sseD, sseE, sseF, sseG, sseJ, sseK2, sseL, steA, steB, steC	100% (n=146)
entB, mig-14, pipB2, sinH and *sseI/srfH*	99.3% (n=145)
sseK1	98.7% (n=144)
sspH2	94.6% (n=138)
grvA	39% (n=58)
gogB	32% (n=48)
rck, spvB, spvC and *spvR*	31% (n=46)
pefB, pefC, pefD and *pefA*	30% (n=44)
shdA	14.4% (n=21)
entE and *sspH1*	4.8% (n=7)

Supplementary Table S6. Data on the informative genes across virulotypes.

Genetic element	Gene	Functional category
SPI-2	*gogB*	Intracellular immune modulation; T3SS effector
SPI-2	*pipB2*	Intracellular transport and vacuolar dynamics
SPI-2	*sseI/srfH*	Systemic dissemination and cell migration
SPI-2	*sseK1*	Immune modulation; NF-κB inhibition
SPI-2	*sspH2*	Ubiquitin ligase effector; intracellular survival
SPI-1/associated island	*sspH1*	T3SS effector; inflammatory modulation
SPI-3 / PhoP-PhoQ regulated	*mig-14* (*pagP-related*)	Resistance to antimicrobial peptides
SPI-RCS/ accessory element	*sinH*	Adhesion; associated with intestinal colonization
CS54 island	*shdA*	Intestinal persistence and colonization
Gifsy-2 prophage-associated	*grvA*	Intracellular survival / systemic virulence
pSLT	*spvB*	Actin ADP-ribosylation; systemic virulence
pSLT	*spvC*	Phosphothreonine lyase; MAPK suppression
pSLT	*spvR*	Transcriptional regulator
pSLT	*rck*	Complement resistance and invasion
pSLT	*pefA*	PEF fimbriae
pSLT	*pefB*	Fimbrial assembly
pSLT	*pefC*	Fimbrial chaperone/usher
pSLT	*pefD*	Fimbrial biogenesis
Enterobactin	*entB*	Siderophore biosynthesis
Enterobactin	*entE*	Enterobactin activation

Notes: SPI, *Salmonella* pathogenicity island; T3SS, type III secretion system; NF-κB, nuclear factor kappa B; PhoP-PhoQ, two-component regulatory system PhoP/PhoQ; RCS, regulator of capsule synthesis; pSLT, *Salmonella* virulence plasmid LT2; PEF, plasmid-encoded fimbriae; MAPK, mitogen-activated protein kinase; ADP, adenosine diphosphate. Gene names are indicated in lowercase italics.

Supplementary Table S7. Contribution (loadings) of variables used in the PCA to each PC.

Variable	PC1	PC2	PC3	PC4	PC5
CFU/mL before stress	-0.443706586	-0.085755768	0.142883678	-0.40044625	-0.685904399
CFU/mL after stress	-0.053877701	0.558538949	0.065934178	0.599785427	-0.456786032
Post-stress recovery	-0.371622757	0.380924908	0.183854846	0.009437153	0.335803072
C. elegans TD_{50}	0.465297952	-0.054086914	0.18357634	0.09636327	0.081204547
% colonization 48 h PI	-0.121222369	-0.142383795	-0.730575901	-0.09533059	-0.046707936
% colonization 96 h PI	-0.429026618	-0.055664097	-0.255174914	0.383334811	0.255003888
CFU/Nematode 24 h PI	0.170083057	-0.550622608	0.061179904	0.46692848	-0.303865558
CFU/Nematode 48 h PI	-0.26432636	-0.285536518	0.551381742	-0.02264874	0.176948112
CFU/Nematode 72 h PI	-0.384786471	-0.352934859	0.033374738	0.309768675	0.103296991

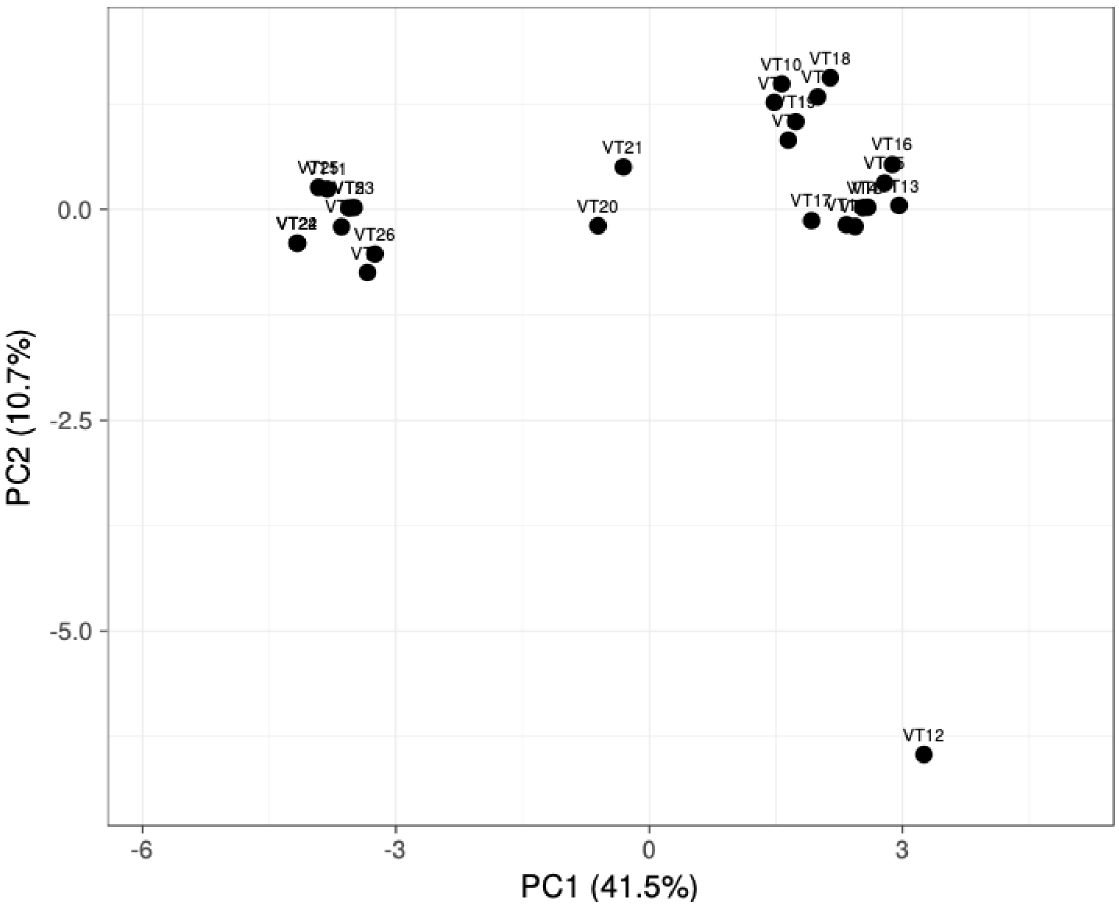

Supplementary Figure S1. Principal Coordinate Analysis of the identified VTs.
